# Defect Engineering in the Porous Salt CAU-55: The Relationship between Cluster Defects and Sorption Properties

**DOI:** 10.1021/acs.langmuir.6c03585

**Published:** 2026-09-30

**Authors:** Bastian Achenbach, Sabrina Grigoletto, Iago Maye, Alejandro Diaz-Marquez, Mohammad Wahiduzzaman, Norbert Stock, Matthias Thommes, Guillaume Maurin

**Affiliations:** † Institute of Inorganic Chemistry, Kiel University Max-Eyth-Str. 2, 24118 Kiel, Germany; ‡ Institut Charles Gerhardt Montpellier, Univ. Montpellier, CNRS, ENSCM, 34293 Montpellier, France; § Institution of Separation Science and Technology, Friedrich-Alexander-Universität Erlangen-Nürnberg (FAU), Egerlandstr. 3, Erlangen 91058, Germany; ∥ Kiel Nano, Surface and Interface Science (KiNSIS) Kiel University Christian-Albrechts-Platz 4, 24118 Kiel, Germany; ⊥ Institut Universitaire de France, Paris 75005, France

## Abstract

Defect engineering has emerged as a powerful strategy to tailor the properties of porous materials. While defect chemistry is well established for metal–organic frameworks (MOFs), the nature and role of defects in porous salts remain unexplored. Here, we report on the synthesis of the porous salt CAU-55-Cl ([Al_24_(OH)_56_(O_2_CCH_3_)_12_]­Cl_4_), which exhibits different sorption properties due to varying defect concentrations that can be achieved by adjusting the molar ratios of the starting materials. Infrared spectroscopy, elemental analysis and thermogravimetric measurements confirmed that the composition of the framework remained unchanged, suggesting that variations in porosity arise from cluster defects (missing Al_24_ clusters) rather than ligand defects (missing acetate ligands) in CAU-55-Cl. Ar (87 K), N_2_ (77 K), CO_2_ (273 K), and H_2_O (298 K) sorption experiments revealed a clear transition from exclusively ultramicroporosity for the defect-free CAU-55-Cl, only accessible for H_2_O and CO_2_, to the presence of additional pores associated with the presence of cluster defects, leading to significantly higher apparent specific surface areas and adsorption capacities with increasing defect concentrations. These experimental findings were further corroborated by molecular simulations, which revealed the geometric characteristics of cluster defects and their influence on adsorption behavior. Grand Canonical Monte Carlo (GCMC) and Widom insertion simulations of Ar as the probe confirmed that missing clusters introduce larger, energetically distinct pore environments, aligning with the observed sorption trends.

## Graphical abstract

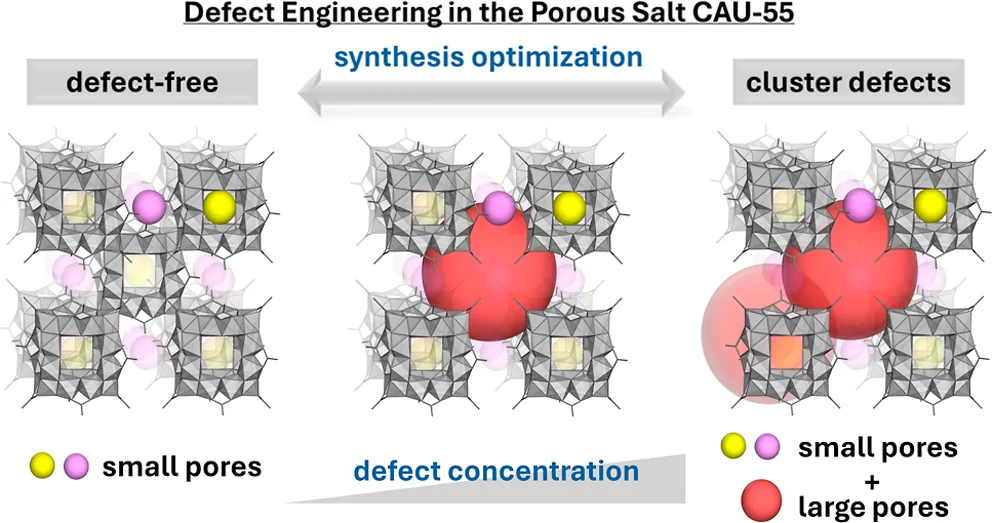

## Introduction

Over the past few decades, porous materials such as metal–organic frameworks (MOFs) and, more recently, porous salts have attracted significant attention.^1–4^ The modular assembly of inorganic building units (IBUs) and organic linkers in MOFs^1,4^ and of charged porous clusters and counterions in porous salts^3,5,6^ leads to a high structural variability and adjustability. A critical aspect influencing the performance of porous materials is the presence of defects, as it can alter their sorption capacities and affinities, catalytic activity, and mechanical stability.^7–10^ It has already been demonstrated that defects in various MOFs can be systematically adjusted through the synthesis process. By carefully adjusting the synthesis conditions, using modulators and varying the molar ratios, it was possible to tailor the type and density of defects in MOFs, thereby modifying their properties. While the formation and characterization of missing linker as well as cluster defects is well established for MOFs,^10,11^ the presence of such defects in porous salts has not, to the best of our knowledge, been discussed. Although it was demonstrated in previous studies on porous salts that synthetic parameters such as the composition of the reaction mixtures and reaction kinetics can be used to tune the composition of the salt or the particle size, these studies did not investigate whether such synthetic variations also lead to structural defects or how they affect the porosity and adsorption behavior.^3^ In principle, there are two types of defects that can occur in porous salts: Ligand defects occur when organic ligands are replaced by other coordinating species, such as water molecules or hydroxide ions. These defects lead to a change in the composition of the porous salt, which can be detected and quantified using spectroscopic methods, titration methods and thermogravimetric analysis.^8,11,12^ Conversely, cluster defects involving missing clusters and their associated counterions can occur without altering the overall material composition, except for the inclusion of guest molecules, making their identification and characterization challenging. One approach for detecting defects and quantifying their concentration involves sorption methods and the correlation of the defect type and content with the change in the type and amount of adsorbed gas or vapor.^9,11,13,14^ This is commonly achieved using probe gases such as N_2_, Ar, CO_2_ and H_2_O.^15^ It has been demonstrated that high-resolution argon (87 K) adsorption isotherms, can be considered as a fingerprint of the adsorbent material, enabling a reliable and detailed evaluation of porosity parameters such as surface area, pore volume, and more importantly pore size distribution.^16,17^ Furthermore, the shape of these isotherms provides valuable insights into adsorption mechanisms and pore-filling behavior, while combined adsorption–desorption analyses reveal possible hysteresis effects.^15^

In parallel, computational methods encompassing quantum chemistry and force field molecular simulations enable a systematic investigation of the wide variety and concentrations of structural defects, as well as their impact on resulting adsorption properties. Previously, systematic studies of missing-linker and cluster defects in prototypical examples of defective frameworks, such as Zr-based MOF-801 and UiO-66, have demonstrated that their progressive incorporation leads to enhancement of hydrophilicity and increased porosity.^18–21^ Typically, changes in hydrophilicity induced by defects are reflected in the water adsorption isotherm by a shift of the pore filling pressure to lower (increase of hydrophilicity) or higher relative pressures (decrease of hydrophilicity) and modification in its multistep profile.^18–21^

For CAU-55-Cl, a porous salt with the composition [Al_24_(OH)_56_(O_2_CCH_3_)_12_]­Cl_4_ containing cationic Al_24_-hydroxide-acetate clusters and chloride ions as counterions,^6,22^ we found significant variations in porosity depending on the synthesis conditions, suggesting the presence of structural defects. Here, we demonstrate that defect formation in CAU-55-Cl can be systematically controlled through synthesis conditions, leading to significant variations in sorption properties. By combining multitechnique experimental characterization (Ar, N_2_, CO_2_, and H_2_O sorption, IR spectroscopy, elemental analysis, thermogravimetry) with molecular modeling (geometric pore analysis and GCMC calculations), we establish that cluster vacancies, not ligand defects, are responsible for the observed porosity changes.

## Experimental Section

### Chemicals

Acetic acid (CH_3_COOH, VWR International GmbH, >99%), aluminum chloride hexahydrate (AlCl_3_·6H_2_O, Alfa Aesar GmbH & Co KG, 99%), methanol (CH_3_OH, Walter-CMP, >97%) and sodium hydroxide (NaOH, Grüssing GmbH, 99%) were commercially obtained and used without further purification.

### Synthesis of CAU-55-Cl with Different Defect Concentrations

Samples of CAU-55-Cl with different defect concentrations were obtained by varying the molar ratios of the starting materials. The molar ratios were varied as follows: 0.5–1.5 equiv of AlCl_3_, 3–7 equiv of CH_3_COOH and 4–6 equiv of NaOH (one equivalent corresponds to 1.44 mmol). Aqueous solutions of AlCl_3_, sodium acetate (CH_3_COOH + NaOH), acetic acid and NaOH were mixed in a 10 mL Duran glass vial, and the reaction volume was kept constant at 8 mL by the addition of water. The reactant solutions were added as aqueous solutions to the glass vials in the following order:

(1) Sodium acetate (NaCH_3_COO; *c* = 2.88 mol/L)
(2) Water (H_2_O)
(3) Acetic acid (CH_3_COOH; *c* = 5.76 mol/L)
(4) Aluminum chloride (AlCl_3_; *c* = 1.44 mol/L)
(5) Sodium hydroxide (NaOH; *c* = 2 mol/L)

Subsequently the reaction vials were sealed, placed in an aluminum block and the reaction mixtures were heated at 100 °C for 20 h under stirring using a heating plate with magnetic stirrer. After cooling to room temperature, the reaction products were separated by centrifugation, washed with water (2·8 mL) and methanol (2·8 mL) and dried at room temperature. The samples are labeled according to the molar ratios of AlCl_3_, CH_3_COOH and NaOH used for the synthesis (AlCl_3_–CH_3_COOH–NaOH). For example, sample 1–4–4 describes a sample that was obtained using the molar ratios AlCl_3_/CH_3_COOH/NaOH = 1:4:4, i.e., with one equivalent of aluminum chloride and four equivalents of acetic acid and NaOH. Section S1 of the Supporting Information provides further details on the exact amounts and types of reagents used, the yields of the reaction products obtained (Table S1), and additional characterization data (Figures S1–S3).

### Powder X-ray Diffraction (PXRD)

The PXRD data were collected in transmission geometry using a STOE Stadi MP and a STOE Stadi P-Combi diffractometer equipped with a MYTHEN 1 K detector and using monochromated Cu Kα1 radiation. The high-throughput powder X-ray diffraction measurements were performed using an xy-stage.

### Elemental Analyses

Elemental analyses of the samples were performed using an Elementar Vario MICRO Cube Elemental Analyzer.

### IR Spectroscopy

IR-spectra of the CAU-55-Cl samples were collected using a Bruker ALPHA-FT-IR A220/D-01 with an ATR-unit.

### Thermogravimetric Measurements

Thermogravimetric measurements were performed on a Linseis STA PT 1000 (airflow = 6 dm^3^/h, heating rate = 8 K/min). The amount of the sample was approximately 25 mg for each sample.

### Sorption Measurements

N_2_ sorption measurements were carried out using a BEL Japan Inc. BELSORP-max, BEL Japan Inc. BELSORP-mini and a BEL Japan Inc. BELSORP-miniX with nitrogen gas at 77 K. High-resolution Argon (87 K) and CO_2_ (273 K) sorption measurements were performed using a high vacuum physisorption analyzer (Autosorb 6100, Anton Paar-QuantaTec, Boynton Beach, Fl 22426). Argon and CO_2_ gases were both purchased from AirLiquide (>99.9999% purity). Argon measurements were performed over a wide range of relative pressure from *p*/*p*_0_ = 10^–6^ to 1. Water sorption measurements were performed at 298 K using a manometric vapor sorption analyzer Vstar from Anton Paar (QuantaTec), where the manifold was kept above at 100 °C. Prior to the measurements, the samples were activated overnight at elevated temperatures (120 °C) under reduced pressure (*p* < 10^–2^ kPa). Subsequently, the integrity and long-range order of the samples after thermal activation and the sorption measurements were confirmed by PXRD measurements after the sorption experiments (Figures S19–S23). Only minor shifts in reflection positions and small changes in the relative reflection intensities are observed. As previously reported for CAU-55-X, these changes are associated with differences in the amount of adsorbed water molecules within the framework rather than structural degradation or defect formation. Since the samples were measured immediately after the sorption experiments, they were expected to be only partially rehydrated under ambient conditions leading to the slight reduction of the lattice parameters, i.e., a smaller unit cell, compared to the as-synthesized materials.^6^ Importantly, no peak broadening or additional reflections are observed, indicating that the framework remains structurally intact throughout the activation and sorption procedures.

## Computational Details

### Construction of Defective CAU-55-Cl Structure Models

To enable a systematic and controlled investigation of defect formation in CAU-55-Cl, a 3 × 3 × 3 supercell was constructed based on the previously reported DFT-optimized crystal structure of this solid.^6^ Each unit cell contains two cage-like clusters of [Al_24_(OH)_56_(O_2_CCH_3_)_12_]^4+^ counterbalanced by eight Cl^–^ anions, resulting in a supercell comprising 54 clusters and 216 chloride ions in total (Figure 1a). This extended model provides sufficient spatial resolution to introduce a broad range of isolated defects while minimizing finite-size artifacts associated with periodic boundary conditions.

**Figure 1:**
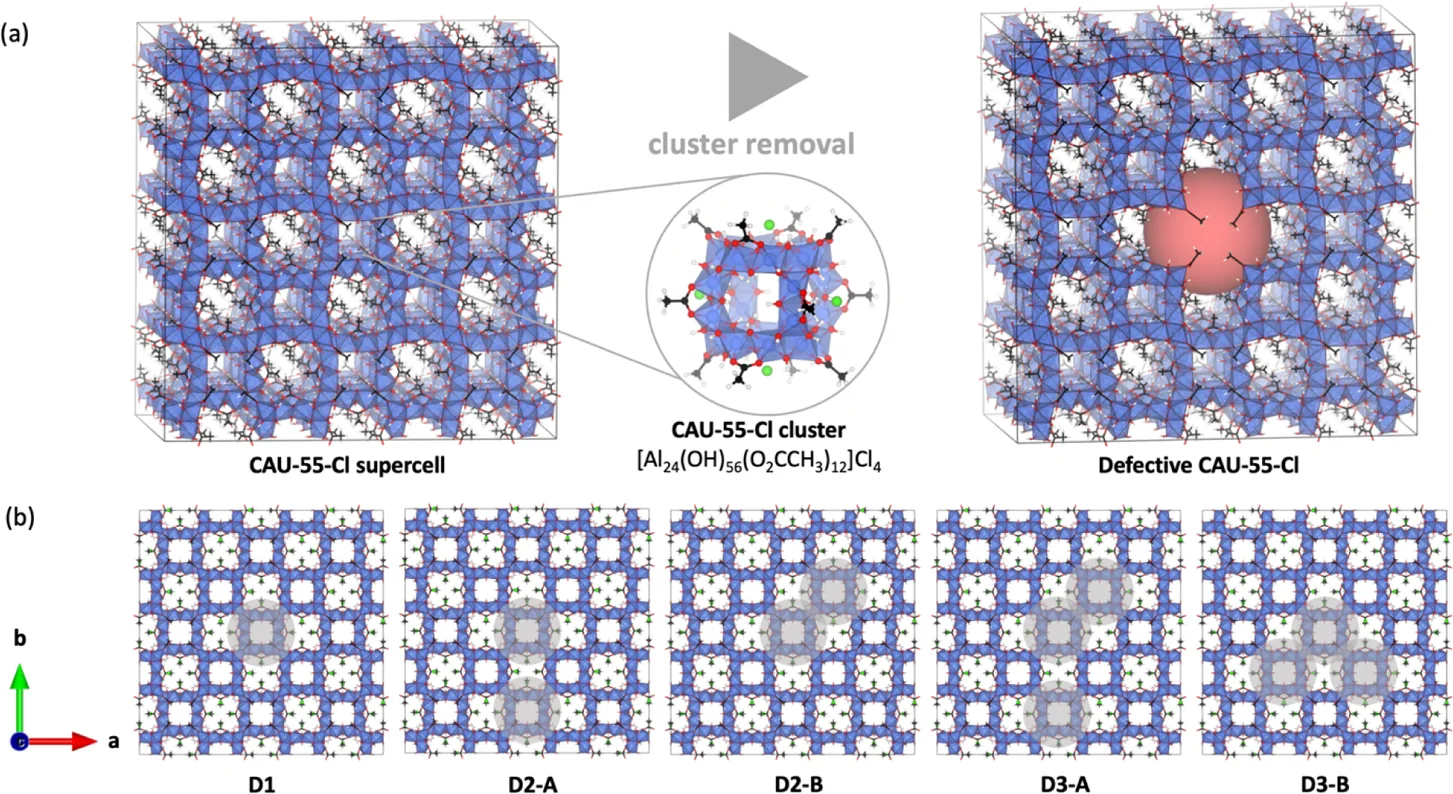
(a) CAU-55-Cl supercell with an inset zoom showing the cage-like cluster [Al_24_(OH)_56_(O_2_CCH_3_)_12_]­Cl_4_ that constitutes the structure, alongside a representation of the defective CAU-55-Cl after the removal of one cluster. (b) Schematic representation of the proposed defects D1 to D3 corresponding to one, two, and three cluster vacancies, respectively.

We created two sets of defective models with complementary purposes:

First, isolated defect models (D1–D3) were constructed to investigate and characterize the intrinsic pore geometry, shape, and size of possible defects within the CAU-55-Cl framework, by selectively removing one to three clusters (Figure 1 and Table 1), along with their associated Cl^–^ ions to preserve overall charge neutrality of the system. For the single-cluster defect (D1), a cluster at the center of the supercell was removed. For the two-cluster case, two configurations were considered: D2-A, where the missing clusters are adjacent and aligned along the same crystallographic axis; and D2-B, where the missing clusters are positioned diagonally (Figure 1b). For the three-cluster defect, two distinct arrangements (D3-A and D3-B) were constructed by combining the spatial motifs of the two-cluster configurations, adjacent (D2-A) and diagonal (D2-B), as depicted in Figure 1b. Notably, the proposed defect configurations are not further geometry optimized. They are representative of a broader range of possible arrangements and do not result in infinite channel-like porosity; rather they give rise to large, localized voids within the framework.

**Table 1:** Structural Characteristics, Textural Properties and Adopted Nomenclature of Defective Structure Models of CAU-55-Cl Considered in This Study^a^

	adopted nomenclature	defect characteristics	density (g/cm^3^)	POAV (cm^3^/g)
isolated defects	D1	single cluster vacancy	1.69	0.21
	D2-A	two adjacent cluster vacancies along same crystallographic axis	1.65	0.23
	D2-B	two diagonal cluster vacancies	1.65	0.23
	D3-A	three cluster vacancies—L shape	1.62	0.24
	D3-B	three cluster vacancies—V shape	1.62	0.24
high defect concentration models	D4	4/54	1.59	0.25
	D6	6/54	1.53	0.27
	D7	7/54	1.49	0.29
	D8	8/54	1.46	0.30
	D9	9/54	1.43	0.32
	D10	10/54	1.40	0.33

a Accessible probe-occupiable volume (POAV) for the models were computed using the Zeo++ software,^23,24^ considering a probe radius of 0.0 Å.

Subsequently, using the same 3 × 3 × 3 supercell, a series of structural models with increasing defect concentrations were constructed by randomly removing clusters from the pristine supercell, representing one among the many possible spatial configurations for each defect concentration. In this context, we constructed additional defective structure models, D*X* (with *X* = 4, 6, 7, 8, 9 and 10), where *X* indicates the number of clusters removed out of the 54 initially present in the pristine supercell. Rather than attempting to reproduce the exact defect arrangement of any specific real sample, these models intended to capture the effect of increasing defect concentration on the simulated adsorption behavior, allowing a semiquantitative estimate of the fraction of missing cages in the experimental samples through comparison with the measured saturation isotherms. All structure models (both nondefective and defective, from D1 to D10) are available as Supporting Information.

### Geometric Characterization of Defect Induced Porosity

The pore structures of all structure models were analyzed using GeoDetect, an *in-house* graph-theory-based code that provides both qualitative visualization and quantitative metrics of porosity (https://github.com/AlejandroDiazMarquez/Geodetect).^25^ The framework was discretized into a 3D grid (0.5 Å spacing), with grid points farther than one probe radius (1 Å) from any atom retained as accessible void space. These points were treated as nodes in a graph network, with edges connecting neighboring nodes within a cutoff distance of 1.0 Å. The eccentricity of each defect defined as the maximum geodesic distance between any two nodes within a connected pore subgraph was computed as a shape-independent pore size descriptor.25 Density and accessible probe-occupiable volume (POAV) for the models were computed using the Zeo++ software,^23,24^ considering a probe radius of 0.0 Å.

### Monte Carlo Adsorption Simulations

Grand canonical Monte Carlo (GCMC) simulations were performed to calculate argon adsorption isotherms at 87 K for both pristine and defective CAU-55-Cl structure models over a pressure range from 1 × 10^–3^ to 1 bar. Additionally, to gain deeper insight into the adsorption energetics, Widom insertion simulations were conducted in three scenarios: (i) a pristine, nondefective structure; (ii) a D1 defective model without accessibility restrictions; and (iii) the same D1 model but with the internal pore spaces of the clusters blocked, thereby preventing intracage adsorption and forcing argon adsorption to occur exclusively within the intercage voids of D1-type defect (i.e., the missing cluster). This approach was devised to assess differences in Ar adsorption enthalpy between the porosities inside the [Al_24_(OH)_56_(O_2_CCH_3_)_12_]^4+^ clusters and the defect-induced missing cage porosity.

In these simulations, host–guest intermolecular interactions were modeled using a Lennard–Jones (LJ) potential. The force–field parameters for both the nondefective and defective CAU-55 framework models were adopted from previous study on this material.^6^ In particular, the force field combined UFF^26^ parameters for Al and Cl atoms with DREIDING^27^ parameters for the remaining atoms, the LJ parameters were kept the same in the pristine and defective models. The LJ parameters for both the framework and argon atoms are provided in Table S4 of the Supporting Information. Dispersion interactions were truncated at a cutoff radius of 12 Å, and Lennard–Jones cross-interactions terms were calculated using Lorentz–Berthelot mixing rules. All simulations were performed using the Complex Adsorption and Diffusion Simulation Suite (CADSS).^28^

## Results and Discussion

To investigate the influence of the synthesis conditions on the defect formation in CAU-55-Cl, the molar ratios of the starting materials were varied between 0.5–1.5 equiv for AlCl_3_, 3–7 equiv for CH_3_COOH and 4–6 equiv for NaOH (Figure 2a). In line with the previous studies,^6,15^ CAU-55-Cl could be obtained over a broad range of molar ratios used for the synthesis. Five representative CAU-55-Cl samples were selected for a more detailed characterization: three samples obtained with varying AlCl_3_ concentrations (0.5 eq., 1.0 eq., 2.0 eq.; samples 0.5–4–4, 1.0–4–4 and 2.0–4–4), one sample synthesized with an excess of NaOH (1–5–6), and one sample obtained with an excess of CH_3_COOH (1–5–4). The samples were labeled according to the molar ratios used in the synthesis. For example, sample 1.0–5–4 describes a sample obtained using one equivalent of AlCl_3_, five equivalents of CH_3_COOH and four equivalents of NaOH. Powder X-ray diffraction (PXRD) data (Figure 2b) confirmed that all five CAU-55-Cl samples were obtained as phase-pure products, as confirmed by the absence of additional reflections. In addition, only minor variations in the crystallinity of the samples were observed. A slight increase in crystallinity with an increasing amount of AlCl_3_ (0.5–4–4 < 1.0–4–4 < 2.0–4–4) can be observed, and when an excess of CH_3_COOH was used compared to NaOH (1.0–5–6 < 1.0–5–4). Furthermore, representative SEM images (Figure S9) reveal a transition from well-defined cubic crystals with smooth surfaces for samples exhibiting low defect concentrations (sample 2–4–4) to particles with rougher particle surfaces and less sharply defined crystal edges for samples with higher defect concentrations (sample 1–5–5).

**Figure 2:**
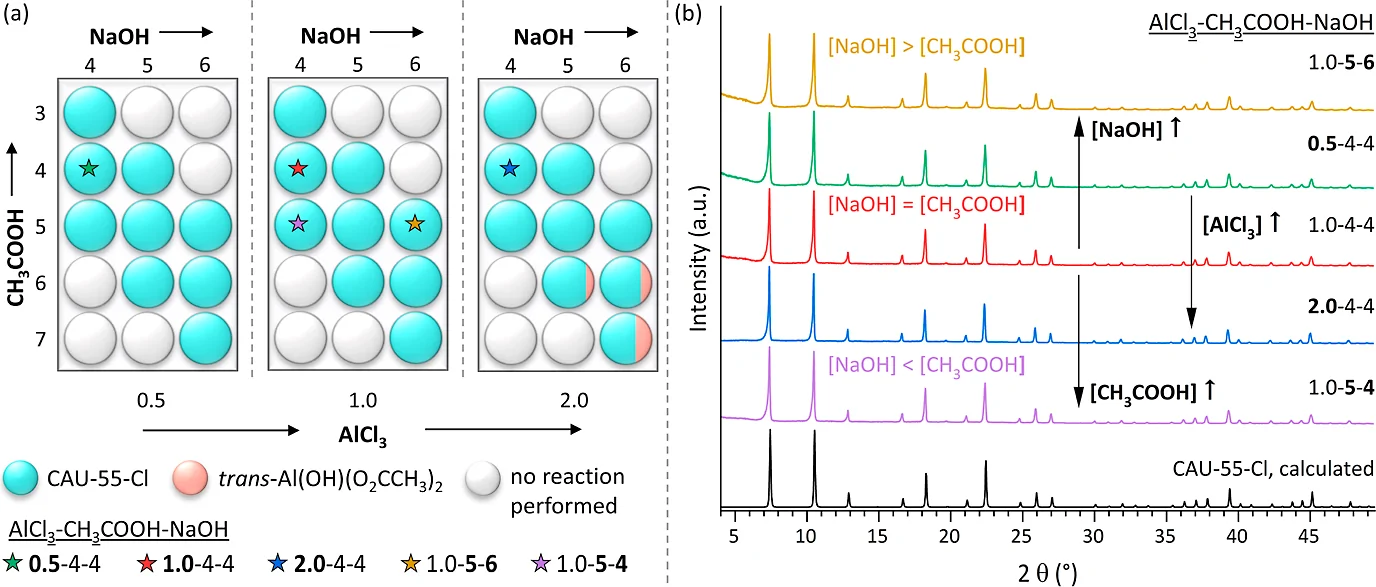
(a) Crystallization diagrams of crystalline phases observed in the chemical system AlCl_3_/CH_3_COOH/NaOH in water. The molar ratios of the starting materials were varied between 0.5 and 2 (AlCl_3_), 3 and 7 (CH_3_COOH) as well as 4 and 6 (NaOH) equivalents. The crystalline phases obtained are color-coded and gray circles represent parameter spaces where no reaction was performed. Samples marked with “★” were used for further characterization. (b) Measured and calculated (black line) PXRD patterns of CAU-55-Cl synthesized using different molar ratios of AlCl_3_, CH_3_COOH and NaOH. The samples are labeled according to the molar ratios used in the synthesis.

IR spectra (Figure 3) were collected for all samples and the associated vibrational bands were identified using literature^29–31^ (Table S2). No differences in the observed bands were found for the different samples, indicating minor or no variation in their composition. This observation was further confirmed by the results of the elemental analysis (Table 2) and the thermogravimetric measurements (Figures S7 and S8 and Table 2), which were used to derive the chemical compositions of the different samples in Table 2. While no difference in the composition of the framework was observed, a significant variation in the amount of adsorbed water molecules within the pores of CAU-55-Cl was found. Specifically, the amount of water decreases with increasing AlCl_3_ concentration used for the synthesis correlating with a decrease in porosity. Importantly, IR spectroscopy, elemental analysis, and thermogravimetric analysis consistently indicate that the chemical composition of the cationic cluster remains unchanged for all investigated samples. If ligand defects (missing acetate ligands) or partial metal deficiency within the Al_24_ clusters had occurred, changes in the Al-to-acetate ratio and thus in the framework composition would be expected. Furthermore, our previous studies of CAU-55-Cl, including solid-state NMR spectroscopy, demonstrated the presence of a single crystallographic aluminum environment, supporting the structural integrity of the Al_24_ cluster as the fundamental building unit.^6^ Instead, the observed differences are limited to the amount of guest molecules within the pores, indicating changes in the accessible pore volume rather than in the framework composition. These observations therefore strongly support the presence of missing-cluster defects rather than ligand defects.

**Figure 3:**
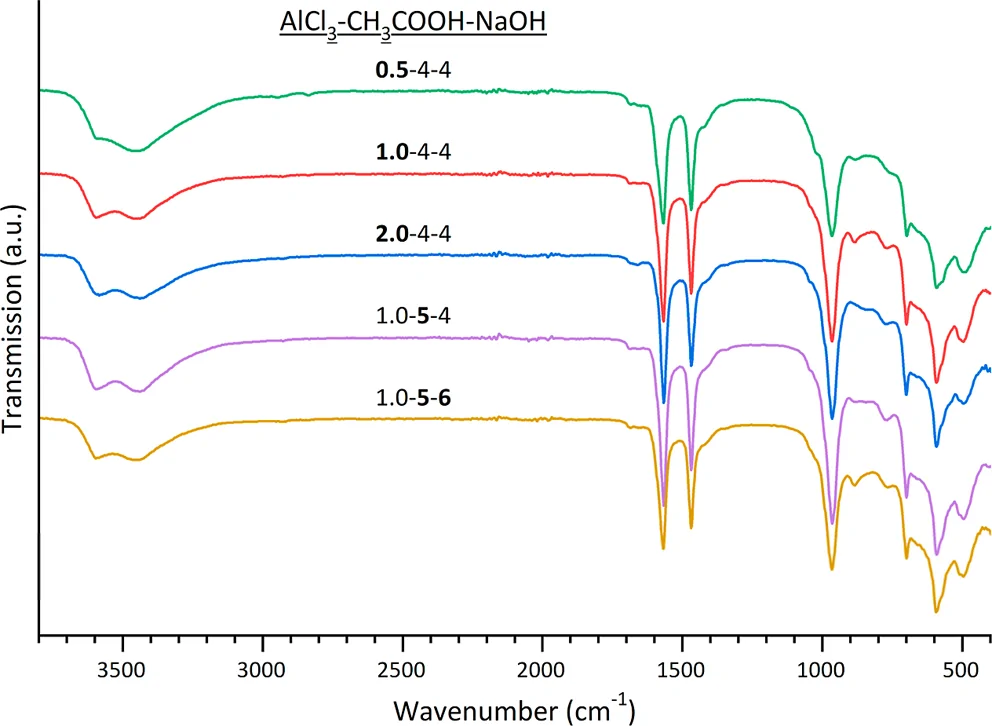
IR spectra of the five different CAU-55-Cl samples. The characteristic vibration bands are assigned in Table S2.

**Table 2:** Results of Elemental Analysis and Weight Losses, Determined by Thermogravimetric Measurements (Figures S4–S8) of the CAU-55-Cl Samples^a^

sample	0.5–4–4	1.0–4–4	2.0–4–4	1.0–5–4	1.0–5–6
chemical composition	[frame]^b^·29H_2_O	[frame]^b^·25H_2_O	[frame]^b^·14H_2_O	[frame]^b^·17H_2_O	[frame]^b^·25H_2_O
C (at %)	9.9 [9.7]	9.7 [9.9]	10.3 [10.7]	10.2 [10.4]	9.6 [9.9]
H (at %)	5.0 [5.1]	4.9 [4.9]	4.5 [4.5]	4.6 [4.6]	5.2 [4.9]
Δ*m*_solvent_ (%)	17.6 [17.6]	15.6 [15.5]	9.3 [9.3]	11.1 [11.1]	15.3 [15.5]
Δ*m*_framework_ (%)	41.3 [41.3]	40.7 [42.2]	48.3 [45.4]	43.1 [44.5]	41.8 [42.3]
*m*_residue_ (%)	41.1 [41.2]	43.7 [42.2]	42.4 [45.3]	45.8 [44.4]	42.9 [42.2]

a The theoretical values assuming the proposed chemical compositions (Table S3) are given in square brackets.

b [Frame] = [Al_24_(OH)_56_(O_2_CCH_3_)_12_]­Cl_4_.

To investigate the structural characteristics of potential cluster vacancies within the CAU-55-Cl framework, we combined computational modeling with high-resolution argon adsorption measurements. Argon at 87 K was selected as the primary characterization probe since, unlike nitrogen, argon lacks a quadrupole moment. Consequently, the resulting adsorption isotherms reflect essentially only the textural properties of the material. This allows for reliable determination of pore size distributions using statistical mechanics-based methods such as nonlocal density functional theory (NLDFT) and methods based on molecular simulations. We first constructed a series of defect-engineered models by systematically removing one to three Al_24_ clusters from a 3 × 3 × 3 supercell of the DFT-optimized CAU-55-Cl crystal structure. These configurations represent localized disruptions in the framework, each introducing voids of distinct shape and size. Graph network analysis using GeoDetect 25 revealed the geometric characteristics of these defects and provided quantitative pore size descriptors. For a single missing cluster (D1), the analysis showed a roughly spherical cavity decorated with protruding void regions corresponding to spaces formerly occupied by acetate groups (Figure 4a,b). The computed eccentricity for D1 of 17.5 Å, is consistent with the distance between hydrogen atoms from acetate groups located at opposite extremities of a Al_24_ cluster (Figure 1a). For two-cluster defects, the D2-A configuration (adjacent clusters along same crystallographic axis) creates interconnected spherical voids, while D2-B (diagonal arrangement) forms a more cylindrical cavity due to removal of cages with acetate groups arranged in a hydrogen-bonded interaction network. This difference arises because, in the D2-B orientation, the removed cages shared a region where their acetate groups were positioned side-by-side, interacting via hydrogen bonding in the pristine structure. Among the D3 type defects, the D3-A structure exhibits a more expanded porosity resulting in eccentricity value that exceeds 30 Å, while D3-B features a more connected, compact porosity with an eccentricity of 26.0 Å. Exhaustive structural details for D2 and D3 defect types are provided in Figure S25.

**Figure 4:**
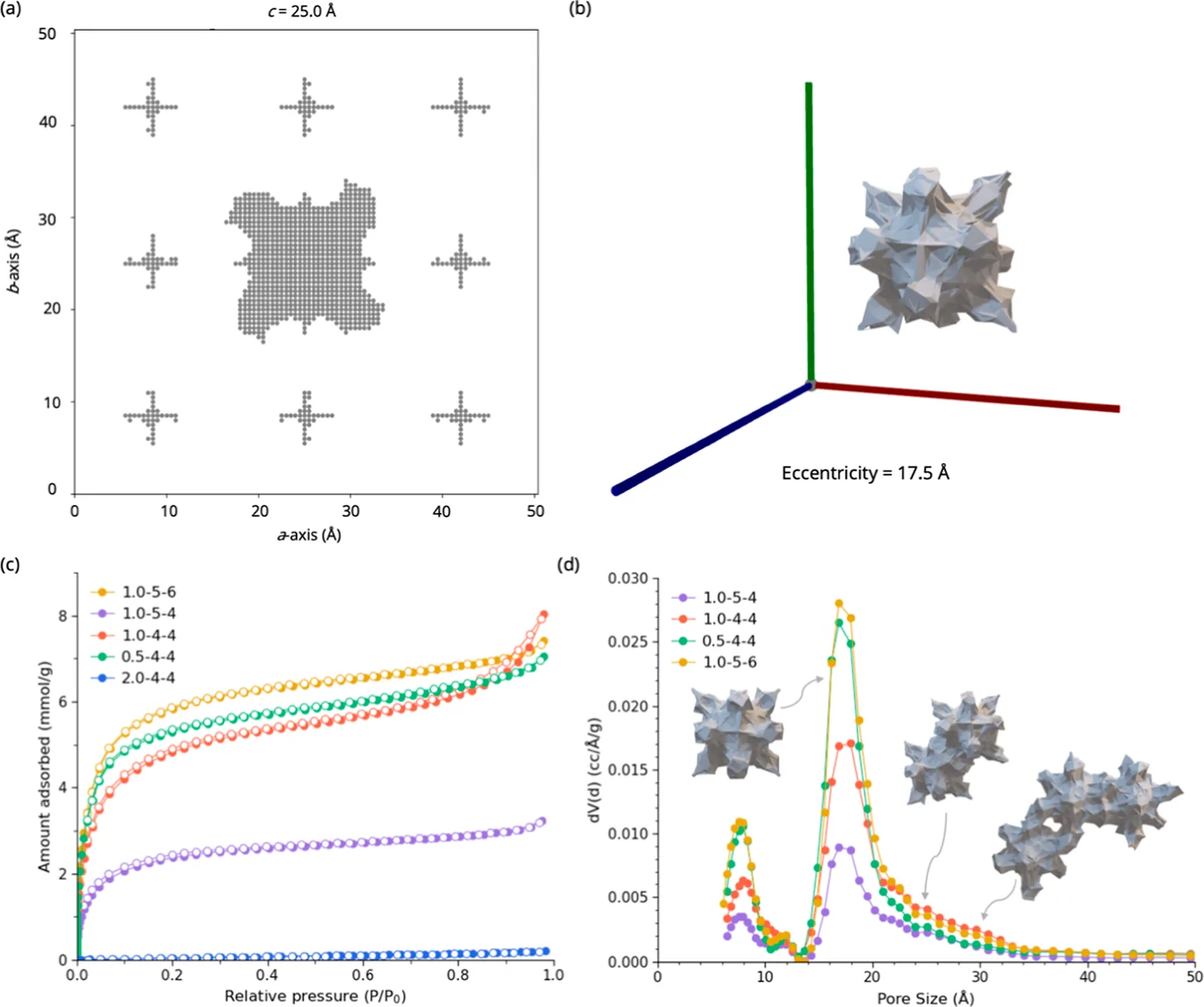
Graph network analysis defective model D1. (a) 2D representation along the *c*-axis (*c* = 25 Å) showing the distribution of pores, defined by the gray dots obtained through graph theory analysis, highlighting the missing-cluster defect and the center of adjacent clusters (b) 3D visualization of the cavity morphology of the missing-cluster defects. (c) Experimental Argon adsorption isotherms and the respective calculated PSD (d) accompanied by indicative isolated defect morphologies. The first peak is likely associated with the cage accessibility or the accessibility of pores between the cages due to partial occupation of the counterion positions, which are shifted from the ideal position (1/2 0 1/2) and (1/2 0 0) to (1/2 *y* 1/2) and (*x* 0 0),^2^ which becomes more pronounced when the defect concentration increases.

Pore size distribution (PSD) analysis of high-resolution Ar adsorption data measured at 87 K (Figure 4c) performed by applying a NLDFT kernel (assuming a spherical/cylindrical pore geometry for the microand mesopores, respectively, and an oxidic surface)^32,33^ revealed excellent agreement with the computational results. The experimental PSD for defective samples showed a dominant peak at 17–18 Å (Figure 4d), in line with the computed eccentricity of 17.5 Å for the D1 defect model. This remarkable size correspondence strongly indicates that single-cluster vacancies are likely the predominant defect type present in CAU-55-Cl (Figure 4b). Notably, even the simplest defect, removal of a single cluster, leads to a drastic change in the available pore volume. This defect generates additional large microporosity, transforming the original micropores (4–5 Å) of the pristine cages into much larger pores starting roughly at 17.5 Å. Samples with higher defect concentrations (0.5–4–4, 1.0–5–6) also exhibited broad/minor PSD features at 24–30 Å, consistent with the computational predictions for D2/D3 multicluster defects. In contrast, sample 2.0–4–4 showed minimal Ar uptake and no larger micropores, confirming the absence of cluster defects in this synthesis condition.

We further performed GCMC simulations of argon adsorption on both pristine and defective models. By comparing simulated Ar uptakes between pristine and D1 model (Figure S26) we quantified the porosity contribution from a single missing cluster. Using this calibration, we proposed higher defect-containing structure models, D4, D6, D7, D8, D9 and D10, containing from 4 to 10 missing clusters per 54-cluster supercell (7–19% defect concentrations, see Table 1), as representative models for investigating the effect of increasing defect concentration and for comparison with the experimental adsorption behavior of the other synthesized samples.

GCMC simulated Ar isotherms for D4–D10 showed good overall agreement with experimental data in the saturation region collected for the synthesized samples (Figure S26). It should be noted that the correspondence between models and experimental samples is established solely through comparison of saturation uptake values, rather than through structural matching. For instance, the simulated sorption uptake at saturation for sample 0.5–4–4 is reproduced by model D10 (10 missing clusters, ∼19% defects), while the adsorption behavior of sample 1.0–5–4 is better reproduced by D4 (∼7% defects). This quantitative agreement between the simulated and experimental saturation uptakes validates both the cluster defect hypothesis and our estimated defect concentrations, although we note that effects arising from the spatial distribution of defects, not explored in the present models, may still influence local adsorption behavior and represent a limitation of this concentration-based approach.

GCMC derived Ar adsorption snapshots collected at different pressure points further enabled elucidation of the progressive adsorption in hieratical pore system of the defective CAU-55-Cl (Figure S27). At low pressure (0.001–0.01 bar), the first Ar atoms preferentially occupy the center of the clusters, while the missing-cluster cavities remain unfilled. At a higher pressure of around 0.05 bar, Ar atoms start to populate the regions of defective porosities, specifically, the spaces formerly occupied by the acetate groups of the cluster, as evidenced by the cavity morphology of the resulting pore network (Figure 4b). Finally, at approximately 0.1 bar, the filling of the missing-cluster cavities becomes evident. Widom insertion calculations quantified this site-specific preference: the computed adsorption enthalpy is −12.0 kJ/mol for the nondefective Al_24_ cluster pores versus only −9.0 kJ/mol for the defect-induced cavities. This 3.0 kJ/mol difference reflects the thermodynamic preferential sittings of Ar in the intracage void at the initial stage of adsorption.

The combined computational-experimental approach thus establishes that (1) single cluster defects create well-defined large micropore cavities of ∼ 17.5 Å in diameter, (2) defects may involve the loss of a single cluster or 2–3 adjacent clusters, (3) single-cluster vacancies (D1) dominate, with multicluster defects (D2/D3) appearing at higher defect levels, and (4) defect concentration ranges from 0% to ∼19% depending on synthesis conditions, consistent with models D4, D9 and D10.

Having established the size and concentration of cluster defects through Ar adsorption, we further investigated how these defects influence accessibility to different probe molecules. Evaluation of the N_2_ sorption data (Figures S10–S14 and Table 3) revealed similar trends in the adsorption behavior of the different CAU-55 samples, as in the Ar sorption experiments at 87 K: no porosity against N_2_ at 77 K was observed for the sample synthesized with the lowest defect concentration (sample 2–4–4), while Type I isotherms were observed for the samples with increasing defect concentrations (0.5–4–4, 1.0–4–4) obtained with decreasing AlCl_3_ concentration. Likewise, with the argon sorption data the highest surface area was observed for the sample prepared with a low AlCl_3_ concentration and a slight excess of NaOH relative to CH_3_COOH (1–5–6). In contrast, despite the kinetic diameter of CO_2_ being very similar to that of argon, all samples exhibited measurable porosity in CO_2_ adsorption experiments at 273 K, which was attributed to the significantly higher measurement temperature. A similar trend to that observed in the N_2_ and Ar sorption properties was observed in the CO_2_ sorption measurements, further indicating an increase in adsorption capacity with increasing defect concentration. Water vapor sorption measurements at 298 K (Figures 5c and S10–S14) provided further evidence of cluster defects in some of the CAU-55 samples, resulting in different pore sizes and pore shapes. The absence of cluster defects in sample 2–4–4 led to a Type I water sorption isotherm (*d*_kin._ ≈ 2.6 Å), with a single adsorption step at low relative pressure (*p*/*p*_0_ < 0.2). This is characteristic for small micropores that cannot be accessed by Ar and N_2_ at their boiling temperatures because of the combination of a larger kinetic diameter (N_2_ ≈ 3.7 Å, Ar ≈ 3.5 Å) and very slow diffusion at the cryogenic temperatures of 87 and 77 K, respectively. In contrast, the water sorption isotherms of the 1.0–5–6 sample showed a two-step adsorption process, with a decrease in the adsorption capacity at low relative pressure, and an additional adsorption step at higher relative pressure (*p*/*p*_0_ ≈ 0.25). The first step of the water sorption isotherm can be related to the adsorption of water molecules inside the Al_24_-clusters, as well as in the pores between the clusters, which are not accessible for Ar and N_2_ at 87 and 77 K, respectively, but are accessible for H_2_O at 298 K and CO_2_ at 273 K. The additional adsorption step observed at higher relative pressures can be attributed to the presence of the larger pores generated by defects of missing Al_24_ clusters. Table 3 summarizes the variations in sorption properties and defect concentrations of the different CAU-55-Cl samples synthesized using different molar ratios of the starting materials.

**Table 3:**
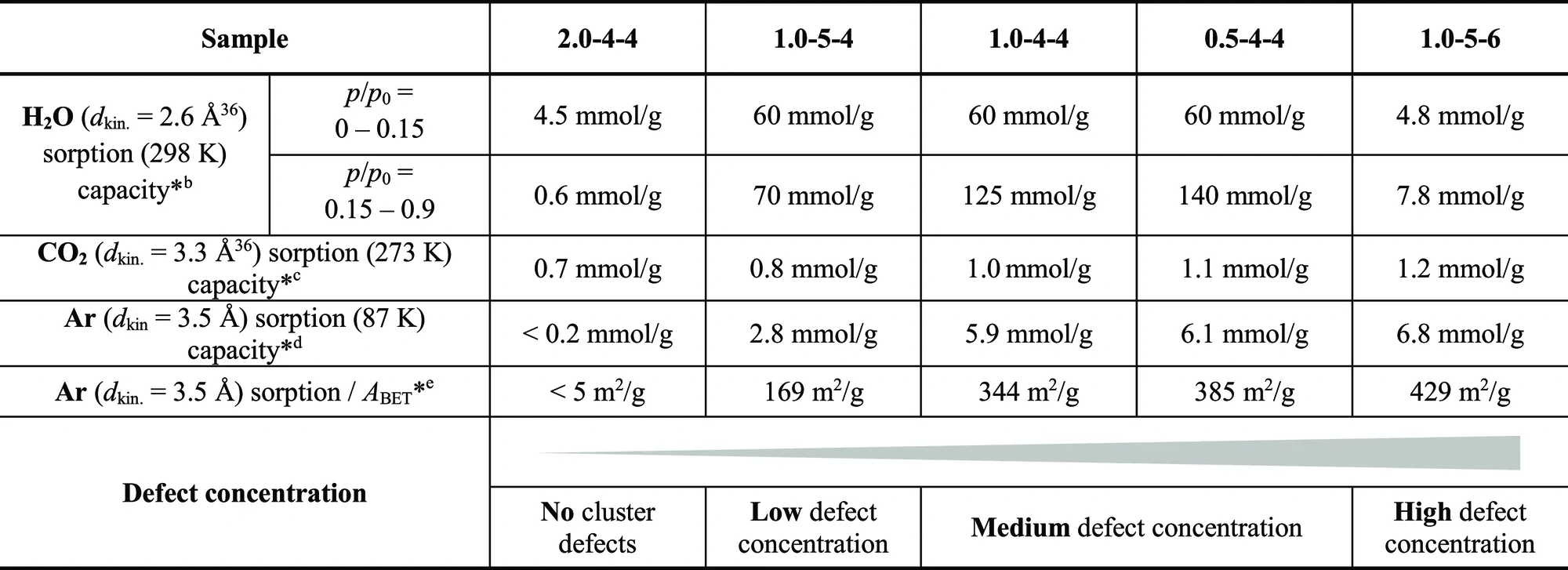
Overview of the Maximum Adsorption Capacities and Apparent Specific Surface Areas Determined from N_2_, Argon and CO_2_ Gas and Water Vapor Sorption Measurements^a^

a For values of samples 1.0–5–4, 1.0–4–4 and 0.5–4–4, the corresponding sorption data is presented in the Supporting Information.

b The H_2_O vapor sorption measurements were carried out at 298 K.

c The CO_2_ gas sorption measurements were carried out at 273 K and the maximum adsorption capacity was determined at *p*/*p*_0_ ≈ 03.

d The Ar gas sorption measurements were carried out at 87 K and the maximum adsorption capacity was determined at *p*/*p*_0_ ≈ 0.7.

e The apparent surface area was determined from the Ar adsorption isotherm employing the methodology by Rouquerol.^34^

**Figure 5:**
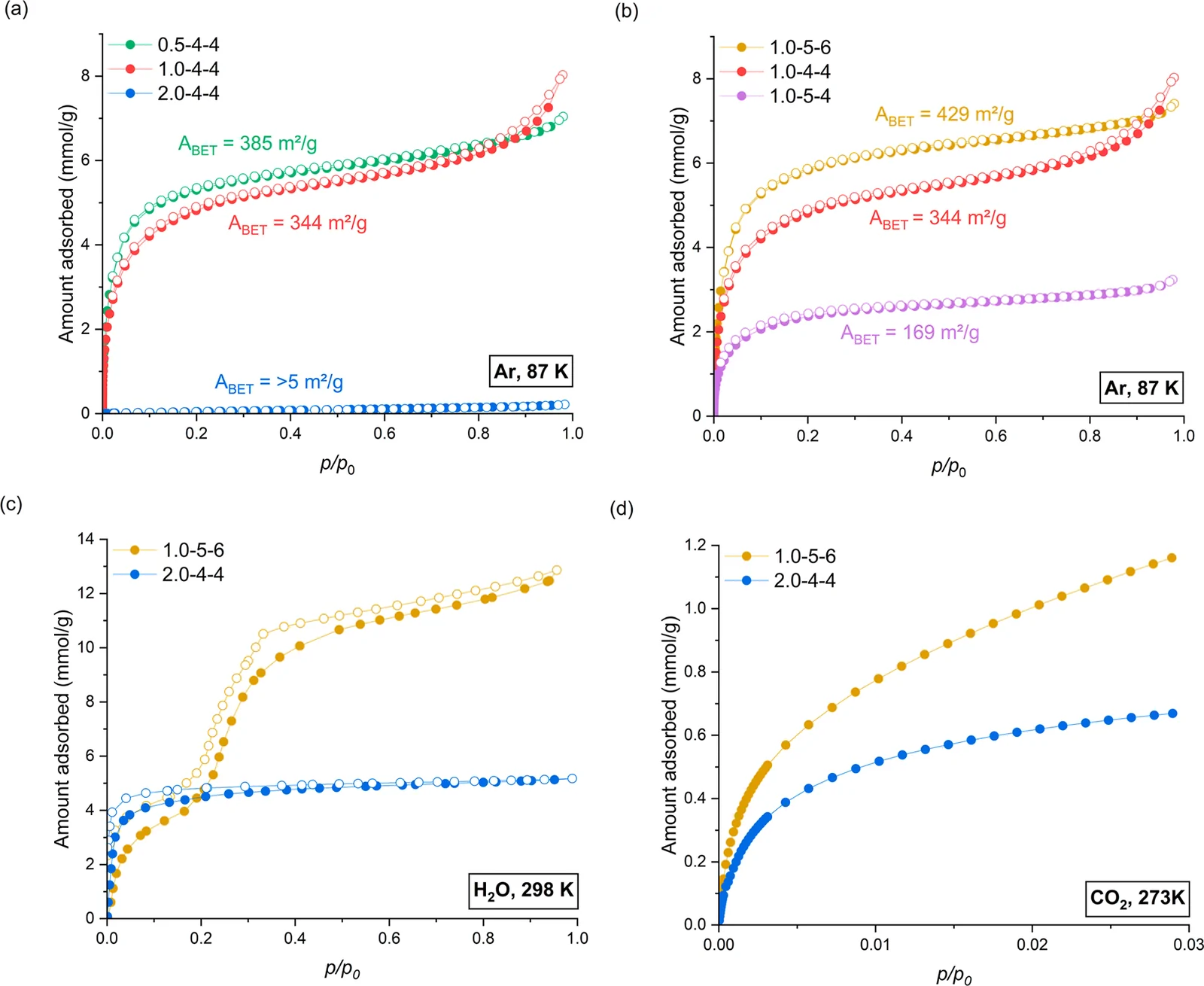
(a,b) Argon sorption isotherms (measured at 87 K) of CAU-55-Cl samples synthesized using different concentrations of AlCl_3_ (a) and varying the molar ratio of CH_3_COOH and NaOH (b). The apparent specific surface area (i.e., BET area) was determined from the Argon sorption isotherm employing the methodology proposed by Rouquerol.^34^ (c,d) Water vapor (c) and CO_2_ gas (d) sorption isotherms of the CAU-55-Cl samples synthesized using different AlCl_3_ concentrations. Ar sorption isotherms in semilog presentation are shown in Figure S24.

An interesting behavior is observed when comparing the CO_2_ adsorption data with the H_2_O adsorption data (exhibiting hysteresis) at low relative pressure (*p*/*p*_0_ < 0.2). In this range, sample 2–4–4 exhibits a higher water uptake than 1–5–6, whereas CO_2_ adsorbs more in 1–5–6 than in 2–4–4. Because both H_2_O and CO_2_ adsorption are performed at similar temperatures and absolute pressure ranges (despite differences in the plotted relative pressure scales), this suggests that the smaller kinetic diameter of water (2.6 Å vs 3.3 Å for CO_2_) allows it to access additional restricted porosity (pore between the clusters) in 2–4–4, which cannot be accessed by the slightly larger CO_2_ molecule. These findings suggest that the process of creating these defects in the CAU-55-Cl material decreases these restrictions toward the clusters (evidenced by Argon and N_2_ being able to access the clusters in the defective samples, see Figure 5a,b), but also reduces the overall amount of these smaller water-only accessible pores.

In order to investigate the origin/contributions of the low-pressure hysteresis in the water sorption isotherm of sample 1.0–5–6, consecutive adsorption/desorption cycles were performed (Figure 6). In the first adsorption–desorption cycle, desorption was measured down to at a relative pressure of *p*/*p*_0_ = 0.13. The second cycle was then immediately started from this same pressure (*p*/*p*_0_ = 0.13), allowing adsorption to proceed, followed by desorption down to approximately the initial pressure of the first cycle (*p*/*p*_0_ ≈ 0.007). As shown in the inset of Figure 6, the second cycle exhibits a significant reduction in the magnitude of the hysteresis compared to the first one. This suggests that the presence of very small pore entrances, which leads to diffusion restrictions even for water adsorption at 298 K, despite its very small kinetic diameter, contributes significantly to the observed low pressure water sorption hysteresis.

**Figure 6:**
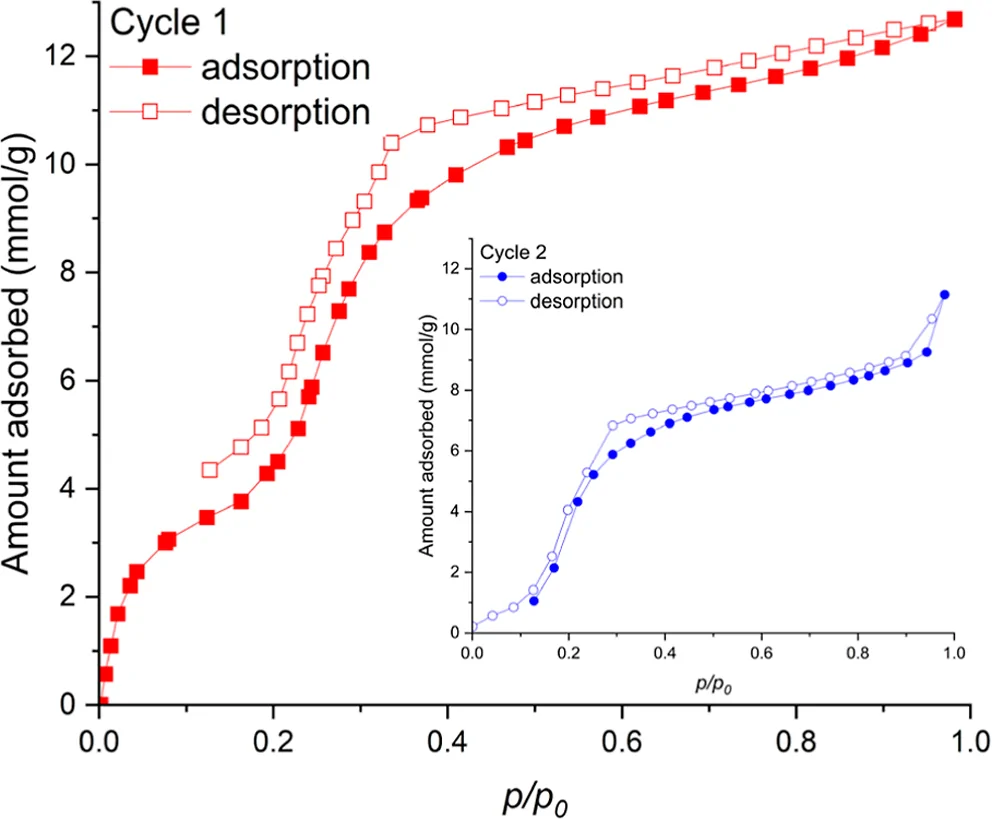
Water sorption isotherms at 298 K obtained during adsorption/desorption cycling of 1.0–5–6. The last desorption point in the first Cycle 1 was taken at *p*/*p*_0_ = 0.13 from which then a consecutive adsorption/desorption cycle was performed (see insert).

Integrating experimental and computational results, we can now present a comprehensive picture of defect-dependent porosity in CAU-55-Cl (Figure 7). The defect-free structure (2.0–4–4) contains exclusively ultramicropores (∼4 Å) within Al_24_ clusters and interstices between clusters. At the temperatures used for adsorption measurements, these pores are accessible to H_2_O (*d*_kin._ ≈ 2.6 Å^35^) and CO_2_ (*d*_kin._ ≈ 3.3 Å^35^) but not Ar (*d*_kin._ ≈ 3.4 Å^35^), explaining the absence of Ar uptake despite significant H_2_O (5.1 mmol/g) and CO_2_ (0.67 mmol/g) adsorption. Lower AlCl_3_ concentration (1.0–4–4) and (0.5–4–4) or an excess of NaOH (1.0–5–6) during synthesis leads to a strong increase of cluster defects, i.e., cluster vacancies concentrations ranging from ∼7% to ∼20%. Each missing Al_24_ unit creates a large spherical micropore (*d* ≈ 17.5 Å) surrounded by protruding voids from exposed acetate groups. These defects are accessible to all probe molecules: The excellent quantitative agreement between computed defect sizes (17.5 Å eccentricity), NLDFT pore sizes (18–19 Å), and Al_24_ cluster dimensions (∼2 nm) clearly suggest that missing clusters are the dominant defects in CAU-55-Cl. To further verify that the observed trends are not limited to the representative samples selected for detailed characterization, the estimated defect concentrations of the additional samples of CAU-55-Cl synthesized using intermediate molar ratios of the starting materials have also been included in Figure 7. These data reveal a continuous evolution of the defect concentration across the investigated parameter space. The representative samples discussed in detail therefore capture the overall trend and cover the complete range from defect-free to highly defective CAU-55-Cl. In addition, three independent syntheses of a representative sample yielded almost identical adsorption isotherms, demonstrating the excellent reproducibility of both the synthesis and the resulting sorption properties, and therefore of the defect concentration (see Figure S17).

**Figure 7:**
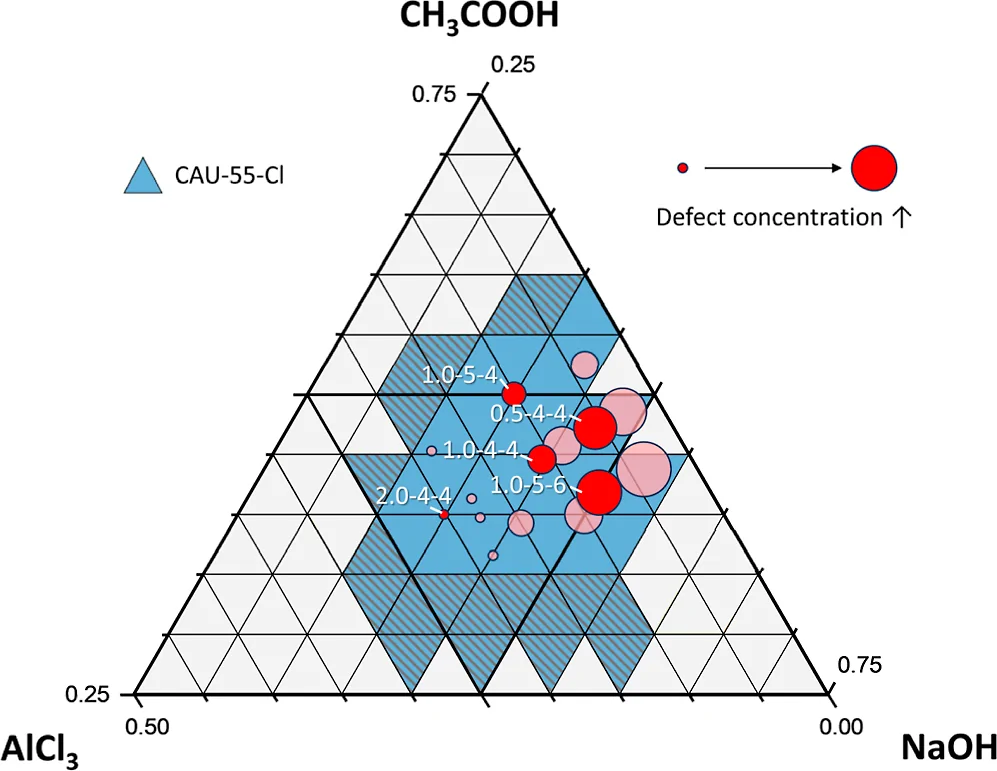
Ternary crystallization diagram of CAU-55-Cl observed in the chemical system AlCl_3_/CH_3_COOH/NaOH for reactions carried out at 100 °C. The blue triangles indicate the parameter spaces in which CAU-55-Cl was obtained and gray-hatched triangles represent parameter spaces where CAU-55 was obtained together with other crystalline phases as a phase mixture (see Table S1). The samples used for a detailed characterization are labeled according to the molar ratios used in the synthesis, with the corresponding defect concentrations illustrated by red dots of different sizes. In addition, light red dots of different sizes indicate the defect concentrations (determined from the apparent specific surface areas of the samples obtained from N_2_ sorption measurements) of other CAU-55-Cl samples examined within this parameter range. The dots are labeled with the corresponding sample numbers in Figure S16.

## Conclusion

This work establishes that porous salts, like MOFs, are amenable to defect engineering for the systematic control of structural imperfections to tailor properties. However, the nature of defects differs: while MOFs commonly exhibit ligand defects due to modulator competition during synthesis, CAU-55-Cl shows exclusively cluster defects that preserve framework stoichiometry. This distinction makes defect identification more challenging but also offers unique opportunities: cluster vacancies introduce large micropores without altering surface chemistry, potentially beneficial for applications requiring hierarchical pore structures.

The identification of simple synthesis parameters (molar ratios of AlCl_3_, NaOH and CH_3_COOH) controlled defect formation provides a straightforward strategy for property optimization. The 17–19% defect concentration achievable in sample 1.0–5–6 represents a significant structural disruption that nevertheless maintains long-range crystallinity, suggesting robust framework stability. Future work will explore whether even higher defect levels are accessible and whether postsynthetic treatments can further modulate defect distributions.

## Supplementary Material



## Data Availability

The CAU-55-Cl structure models as well as the results of the graph network porosity analysis are available at: https://github.com/sabrinagri/supporting-info.

## Notes

Additional experimental details, detailed synthesis procedures, synthesis yields, PXRD data, spectroscopic characterization details, thermogravimetric analyses, chemical compositions, additional gas and vapor sorption measurements, SEM images, computational details (force field definitions, graph network porosity analysis, GCMC adsorption simulations), structural models (PDF)
