# Supplementary material for: Defect Engineering in the Porous Salt CAU-55: The Relationship between Cluster Defects and Sorption Properties
Source: Langmuir. 2026 Sep 30;42(40):29794–804. doi: 10.1021/acs.langmuir.6c03585 (PMC13674237; doi:10.1021/acs.langmuir.6c03585)
Supplement: Supplementary file 1 [file la6c03585_si_001.pdf]

Supporting Information

Defect Engineering in the Porous Salt CAU-55: The Relationship Between Cluster Defects and Sorption Properties

Bastian Achenbach[a], Sabrina Grigoletto[b], Iago Maye,[c] Alejandro Diaz-Marquez[b], Mohammad Wahiduzzaman[b], Norbert Stock*[a,d], Matthias Thommes,*[c] Guillaume Maurin*[b,e]

[a] B. Achenbach, Prof. Dr. N. Stock*
Institute of Inorganic Chemistry
Kiel University
Max-Eyth-Str. 2, 24118 Kiel, Germany
E-mail: stock@ac.uni-kiel.de

[b] S. Grigoletto, Dr. A. Diaz-Marquez, Dr. M. Wahiduzzaman, Prof. Dr. G. Maurin*
Institut Charles Gerhardt Montpellier
Univ. Montpellier, CNRS, ENSCM
34293 Montpellier, France
E-mail: guillaume.maurin1@umontpellier.fr

[c] I. Maye, Prof. Dr. M. Thommes*
Institution of Separation Science and Technology
Friedrich-Alexander-Universität Erlangen-Nürnberg (FAU)
Egerlandstr. 3, Erlangen 91058, Germany
E-mail: matthias.thommes@fau.de

[d] Prof. Dr. N. Stock
Kiel Nano, Surface and Interface Science (KiNSIS)
Kiel University
Christian-Albrechts-Platz 4, 24118 Kiel, Germany

[e] Prof. Dr. G. Maurin
Institut Universitaire de France
France

Table of Contents

Table of Contents 2

1. Synthesis 3

 1.1. Synthesis of the CAU-55-Cl samples 3

2. Spectroscopic characterization, elemental analyses and thermal properties 6

 2.1. IR spectroscopy 6

 2.2. Thermogravimetric measurements 7

 2.3. Compositions of the CAU-55-Cl samples 9

3. Sorption properties 10

4. Computational Details 17

 4.1. Force field definitions 17

 4.2. Graph network porosity analysis 18

 4.3. GCMC adsorption isotherms and adsorption mechanism 19

5. References 20

1. Synthesis

1.1. Synthesis of the CAU-55-Cl samples

Samples of CAU-55-Cl with different defect concentrations were obtained by varying the molar ratios of the starting materials. The molar ratios were varied as follows: 0.5 – 1.5 equivalents of $AlCl_3$, 3 – 7 equivalents of CH_3COOH and 4 – 6 equivalents of NaOH (one equivalent corresponds to 1.44 mmol). Aqueous solutions of $AlCl_3$, sodium acetate, acetic acid and NaOH were mixed in a 10 mL glass vial, and the reaction volume was kept constant at 8 mL by the addition of water. The reactant solutions were added to the glass vials in the following order:

1) Sodium acetate (CH_3COONa; c = 2.88 mol/L)
2) Water (H_2O)
3) Acetic acid (CH_3COOH; c = 5.76 mol/L)
4) Aluminum chloride ($AlCl_3$; c = 1.44 mol/L)
5) Sodium hydroxide (NaOH; c = 2 mol/L)

Subsequently the reaction vials were sealed, placed in an aluminum block and were heated at 100 °C for 20 h under stirring using a heating plate with magnetic stirrer (*MR Hei-Tec, Heidolph*). After cooling to room temperature, the reaction products were separated by centrifugation (*Allegra 64R, Beckman Coulter)*, washed with water (2 · 8 mL) and methanol (2 · 8 mL) and dried at room temperature. The amounts of the starting materials used for the reactions, the reaction numbers and the yields of the different reactions are listed in Table S1. Figure 2b in the main manuscript and Figures S1 – S3 contain the PXRD data of the different CAU-55-Cl samples obtained.

Table S1. List of starting materials employed for the synthesis of CAU-55-Cl samples with different defect concentrations. The samples are labelled according to the molar ratios used in the synthesis. For example, sample 1-3-4 describes a sample that was obtained with one equivalent of $AlCl_3$, three equivalents of CH_3COOH and four equivalents of NaOH. The yields (based on Al^{3+}) of the CAU-55 samples were calculated using the chemical composition determined for the sample 1.0-4-4(C2): $[Al_{24}(OH)_{56}(O_2CCH_3)_{12}]Cl_4 \cdot 25\ H_2O$ (M = 2900.7 g/mol). One equivalent of $AlCl_3$ corresponds to 1.44 mmol. The reaction vials placed in an aluminum block and were heated at 100 °C for 20 h under stirring using a heating plate.

Synthesis-No.	**molar ratios** (eq.)			***V***					Obtained crystalline phase(s)	Yield (mg, %)
	$AlCl_3$	CH_3COOH	NaOH	$AlCl_3$[a] (µL)	CH_3COONa[b] (µL)	CH_3COOH[c] (µL)	NaOH[d] (µL)	H_2O (µL)		
0.5-3-4 (A1)		3			1500	/	720	5280	CAU-55-Cl	76.7 mg (88 %)
0.5-4-4 (A2)		4	4		2000	/	/	5500	CAU-55-Cl	63.4 mg (73 %)
0.5-5-4 (A3)		5			2000	250	/	5250	CAU-55-Cl	70.6 mg (81 %)
0.5-4-5 (A4)		4			2000	/	720	4780	CAU-55-Cl	78.3 mg (90 %)
0.5-5-5 (A5)	0.5	5	5	500	2500	/	/	5000	CAU-55-Cl	80.9 mg (93 %)
0.5-6-5 (A6)		6			2500	250	/	4750	CAU-55-Cl	77.5 mg (89 %)
0.5-5-6 (B1)		5			2500	/	720	4280	CAU-55-Cl	48.8 mg (56 %)
0.5-6-6 (B2)		6	6		3000	/	/	4500	CAU-55-Cl	75.8 mg (87 %)
0.5-7-6 (B3)		7			3000	250	/	4250	CAU-55-Cl	81.7 mg (94 %)
1.0-3-4 (C1)		3			1500	/	720	4780	CAU-55-Cl	167 mg (96 %)
1.0-4-4 (C2)		4	4		2000	/	/	5000	CAU-55-Cl	168 mg (97 %)
1.0-5-4 (C3)		5			2000	250	/	4750	CAU-55-Cl	161 mg (93 %)
1.0-4-5 (C4)		4			2000	/	720	4280	CAU-55-Cl	152 mg (83 %)
1.0-5-5 (C5)	1.0	5	5	1000	2500	/	/	4500	CAU-55-Cl	166 mg (95 %)
1.0-6-5 (C6)		6			2500	250	/	4250	CAU-55-Cl	153 mg (87 %)
1.0-5-6 (D1)		5			2500	/	720	3780	CAU-55-Cl	167 mg (96 %)
1.0-6-6 (D2)		6	6		3000	/	/	4000	CAU-55-Cl	154 mg (89 %)
1.0-7-6 (D3)		7			3000	250	/	3750	CAU-55-Cl	162 mg (93 %)
2.0-3-4 (E1)		3			1500	/	720	3780	CAU-55-Cl	19.8 mg (6 %)
2.0-4-4 (E2)		4	4		2000	/	/	4000	CAU-55-Cl	29.7 mg (9 %)
2.0-5-4 (E3)		5			2000	250	/	3750	CAU-55-Cl	30.7 mg (9 %)
2.0-4-5 (E4)		4			2000	/	720	3280	CAU-55-Cl	219 mg (63 %)
2.0-5-5 (E5)		5	5		2500	/	/	3500	CAU-55-Cl	210 mg (60 %)
2.0-6-5 (E6)	2.0	6		2000	2500	250	/	3250	CAU-55-Cl + *trans*-$Al(OH)(O_2CCH_3)_2$	/
2.0-5-6 (F1)		5			2500	/	720	2780	CAU-55-Cl	335 mg (96 %)
2.0-6-6 (F2)		6	6		3000	/	/	3000	CAU-55-Cl + *trans*-$Al(OH)(O_2CCH_3)_2$	/
2.0-7-6 (F3)		7			3000	250	/	2750	CAU-55-Cl + *trans*-$Al(OH)(O_2CCH_3)_2$	/

[a] c_{AlCl3} = 1.44 mol/L; [b] $c_{CH3COONa}$ = 2.88 mol/L; [c] $c_{CH3COOH}$= 5.76 mol/L; [d] c_{NaOH} = 2.0 mol/L

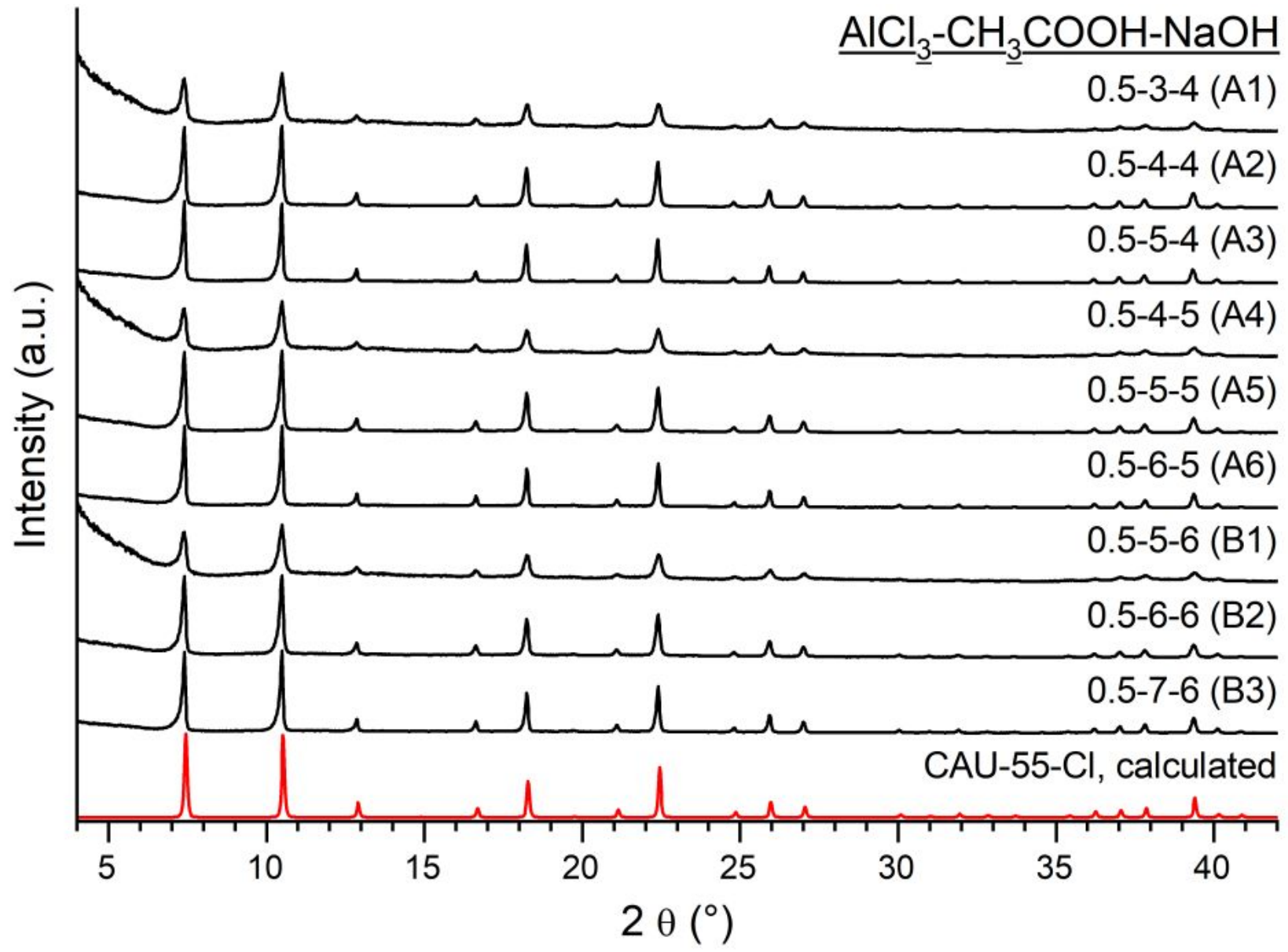

Figure S1. Measured (black line) and calculated (red line) PXRD patterns of CAU-55-Cl samples synthesized using different molar ratios of CH_3COOH and NaOH, while keeping the $AlCl_3$ concentration constant at **0.5 eq**.

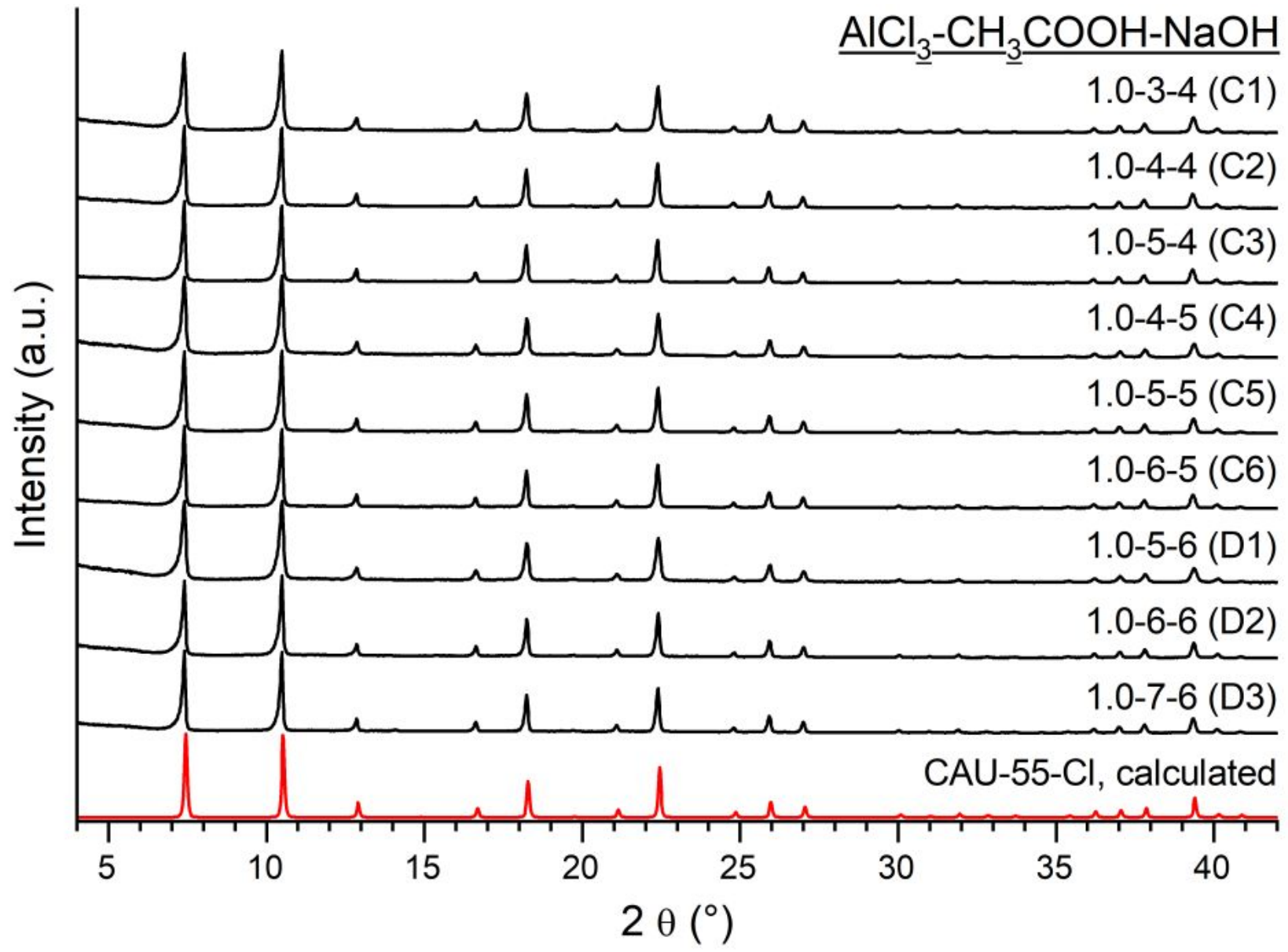

Figure S2. Measured (black line) and calculated (red line) PXRD patterns of CAU-55-Cl samples synthesized using different molar ratios of CH_3COOH and NaOH, while keeping the $AlCl_3$ concentration constant at **1.0 eq**.

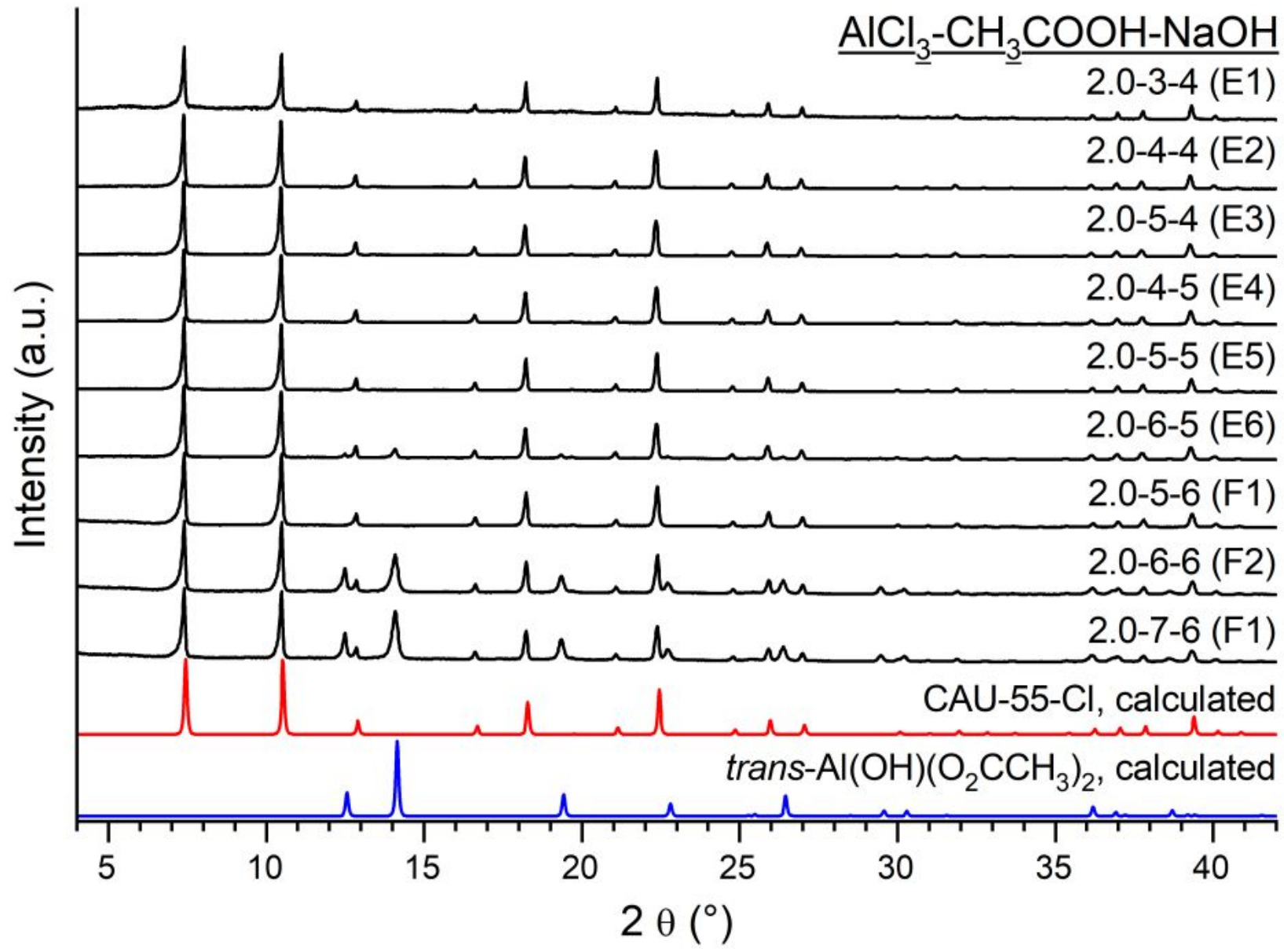

Figure S3. Measured (black line) and calculated (red line) PXRD patterns of CAU-55-Cl samples synthesized using different molar ratios of CH_3COOH and NaOH, while keeping the $AlCl_3$ concentration constant at **2.0 eq**.

2. Spectroscopic characterization, elemental analyses and thermal properties

2.1. IR spectroscopy

IR-spectra of the CAU-55-Cl samples with different defect concentrations are shown in Figure 3 of the main article. The characteristic vibration bands are assigned in Table S2.[1–3]

Table S2. Assignment of the vibrational bands of the CAU-55-Cl samples identified in the IR spectra (Figure 3).

**Vibration** (cm^{-1})	**0.5-4-4 (A2)**	**1.0-4-4 (C2)**	**2.0-4-4 (E2)**	**1.0-5-4 (C3)**	**1.0-5-6 (D1)**
ν_{as}(OH) [H_2O]	3100-3700				
ν_{as}(C-H) [CH_2; CH_3]	2944	/	/	/	2930
ν_s(C-H) [CH_2; CH_3]	2840	2835	2835	2835	2835
ν_{as}(C=O) [CO_2^-]	1570	1565	1565	1565	1570
ν_s(C=O) [CO_2^-]	1470				
ν_s(C-C-O) [CO^{2-}]	965	965	965	965	968
δ(C-H) [CH_3]	698	700	702	701	698

2.2. Thermogravimetric measurements

Thermogravimetric measurements (Figure S4– S8, Table 2) were carried out to determine the solvent content, the thermal stability and the differences in the composition of the CAU-55-Cl samples. The thermogravimetric measurements were performed on a Linseis STA PT 1000 (airflow = 6 dm^3/h, heating rate = 8 K/min). The sample amount was approximately 25 mg for each sample.

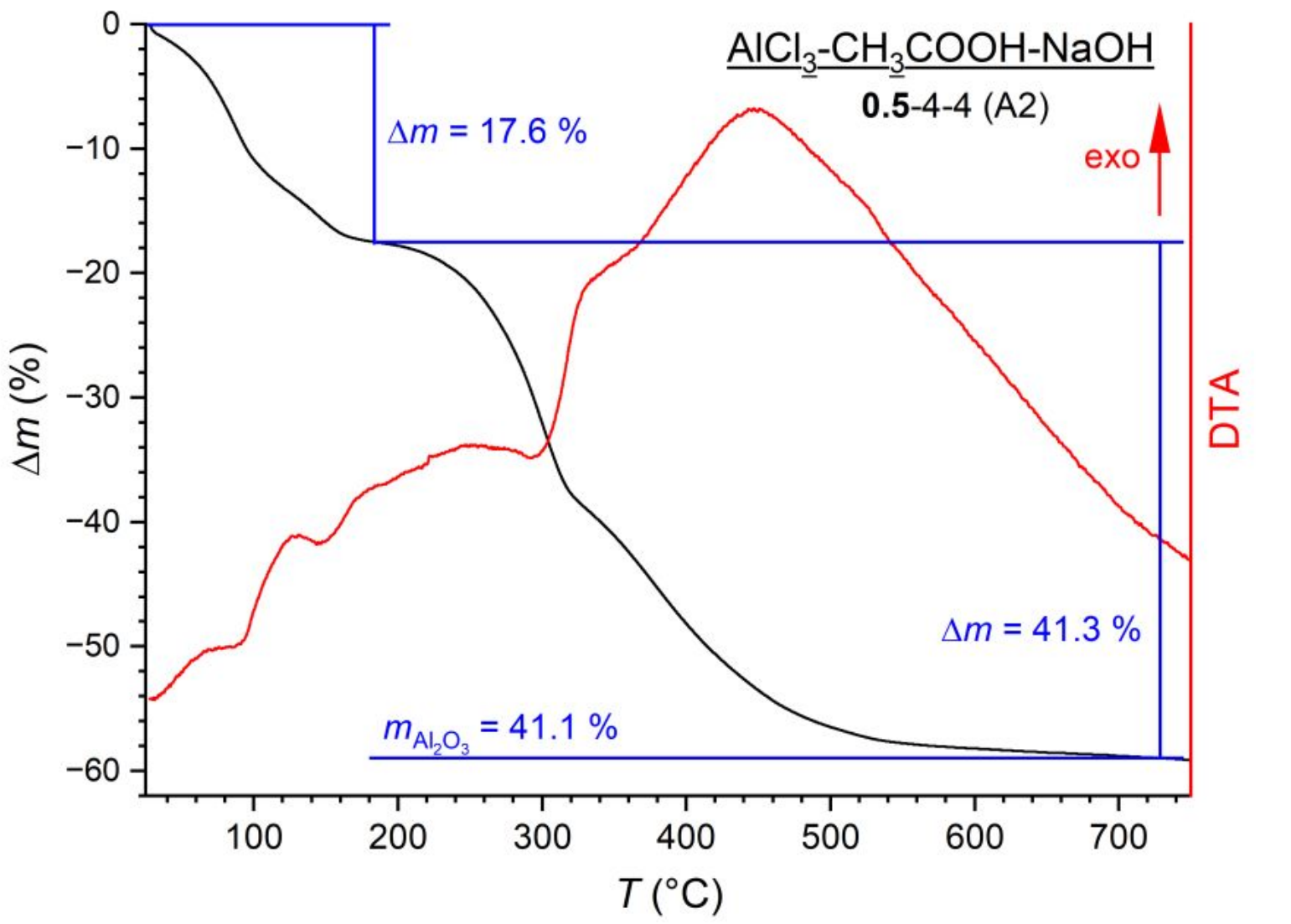

Figure S4. Thermogravimetric curve (black), DTA curve (red) and decomposition steps (blue) of CAU-55-Cl (0.5-4-4 (A2)) measured with a heating rate of 8 K/min in air atmosphere.

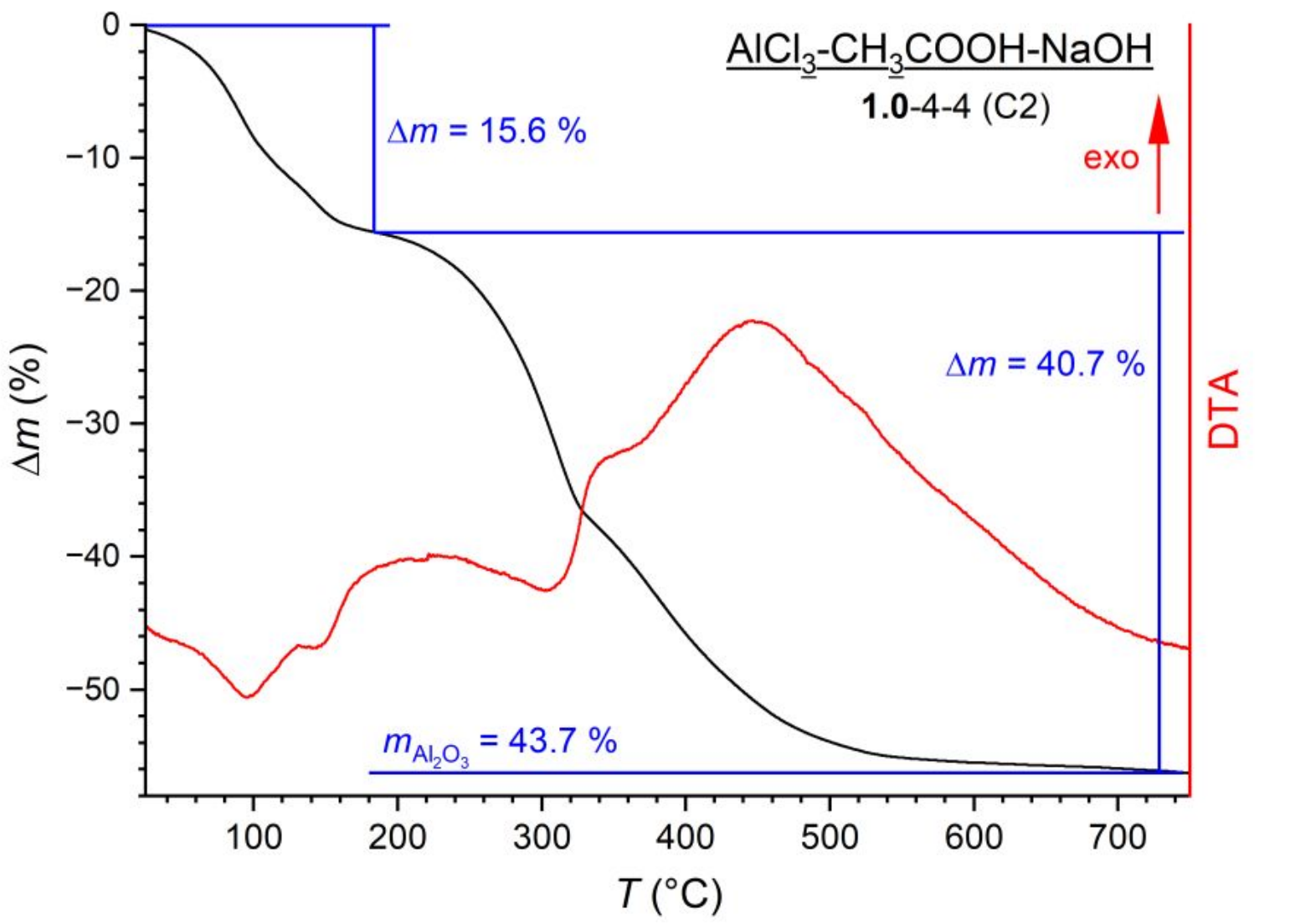

Figure S5. Thermogravimetric curve (black), DTA curve (red) and decomposition steps (blue) of CAU-55-Cl (1.0-4-4 (C2)) measured with a heating rate of 8 K/min in air atmosphere.

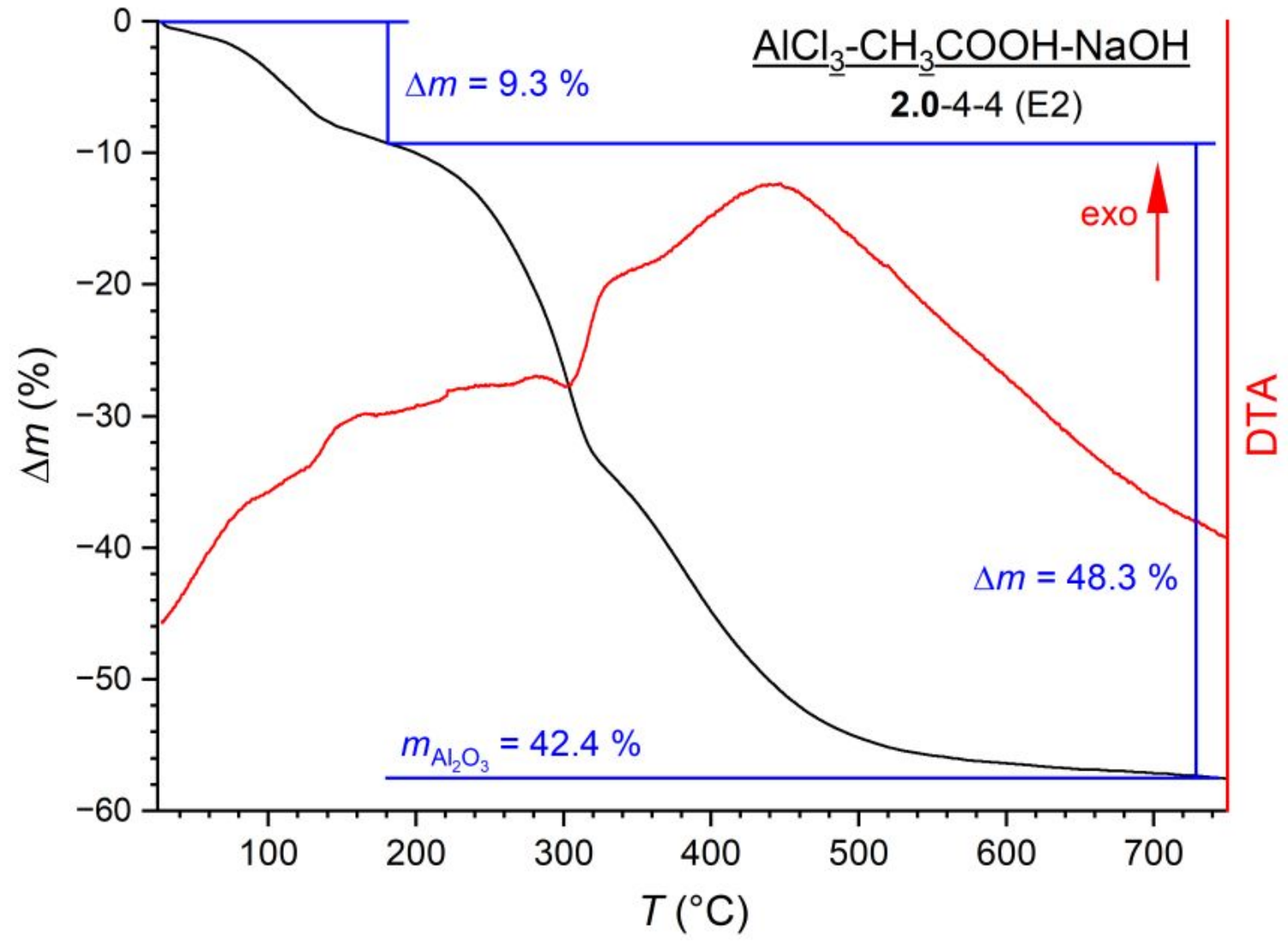

Figure S6. Thermogravimetric curve (black), DTA curve (red) and decomposition steps (blue) of CAU-55-Cl (2.0-4-4 (E2)) measured with a heating rate of 8 K/min in air atmosphere.

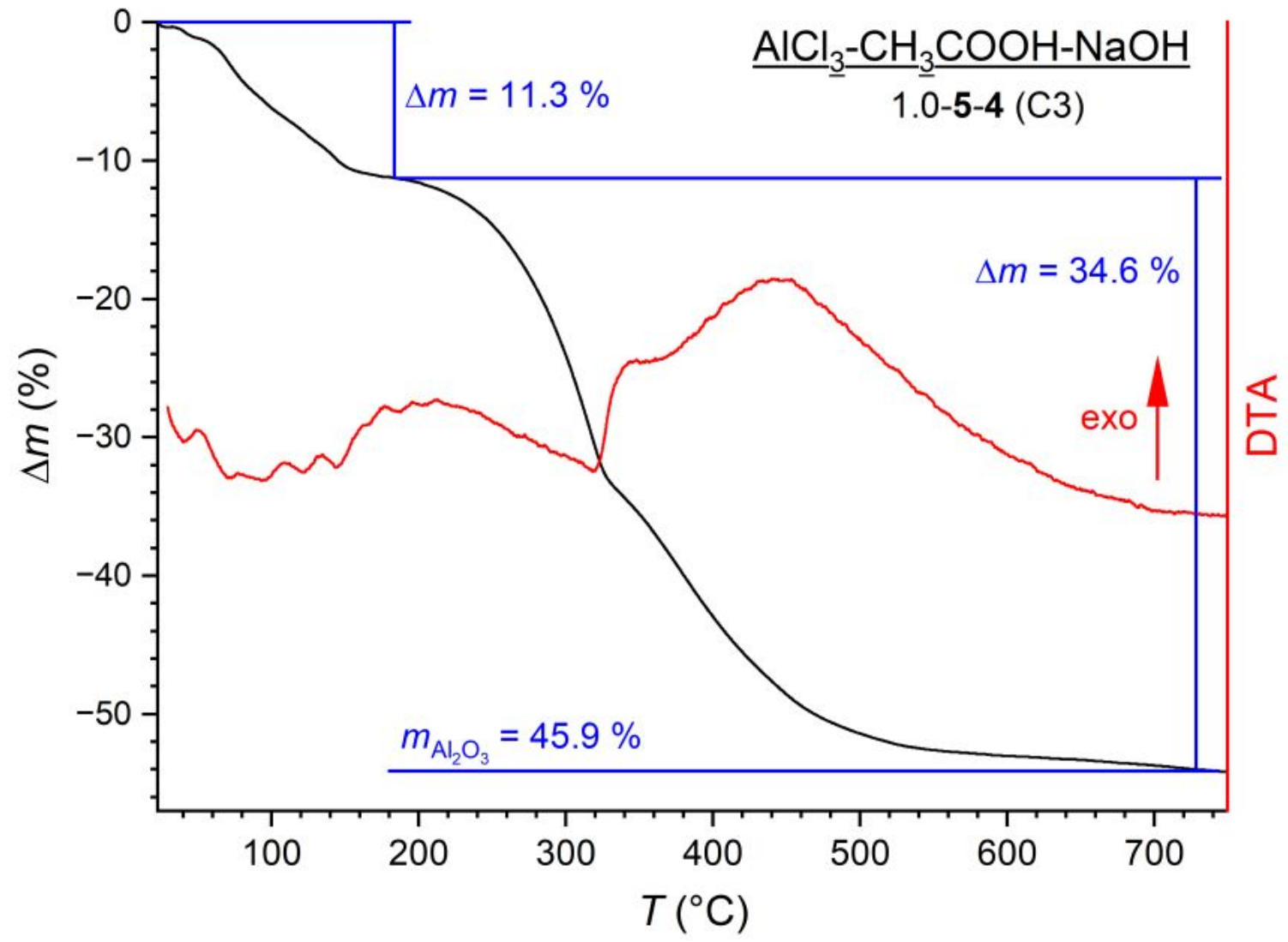

Figure S7. Thermogravimetric curve (black), DTA curve (red) and decomposition steps (blue) of CAU-55-Cl (1.0-5-4(C3)) measured with a heating rate of 8 K/min in air atmosphere.

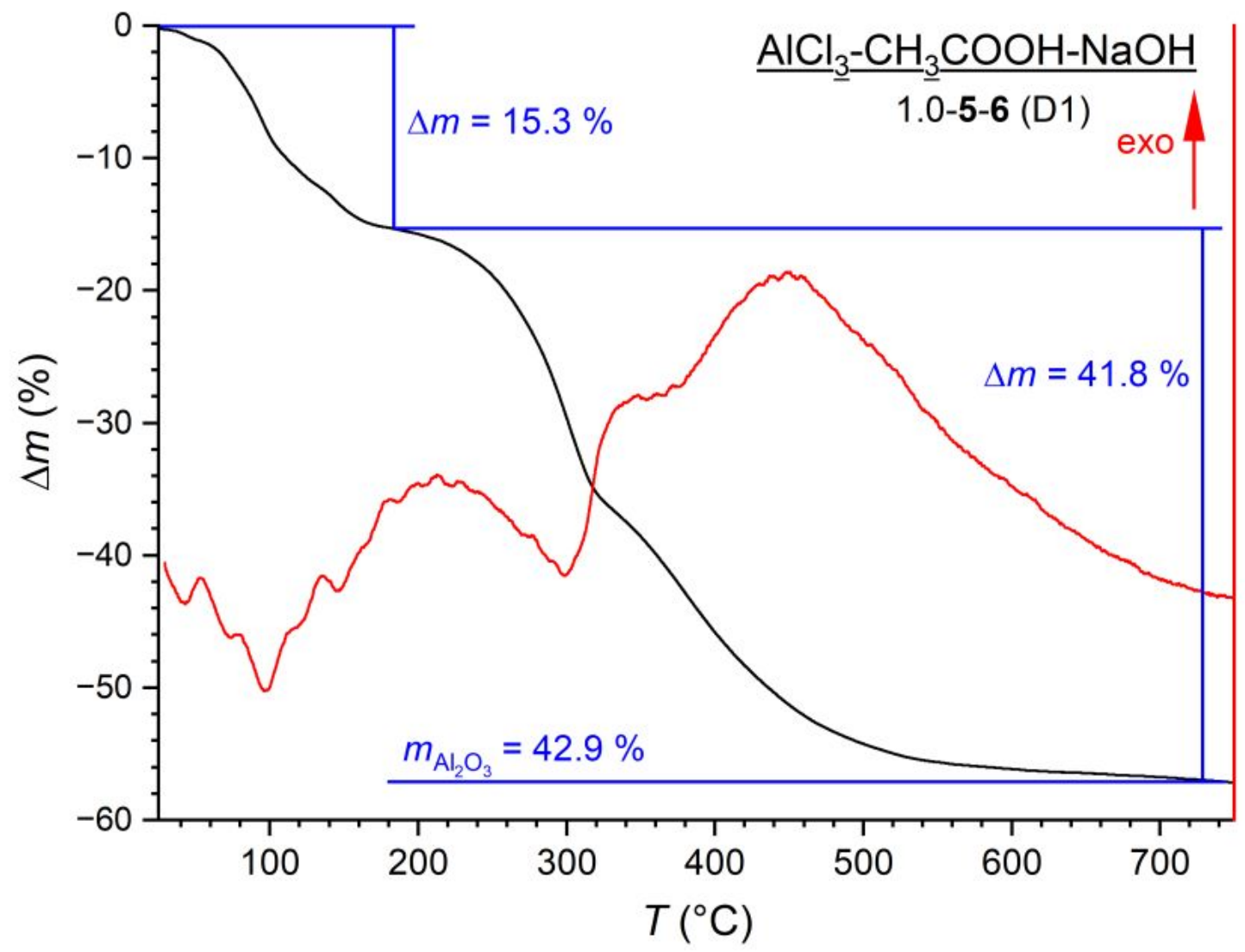

Figure S8. Thermogravimetric curve (black), DTA curve (red) and decomposition steps (blue) of CAU-55-Cl (0.5-5-6(D1)) measured with a heating rate of 8 K/min in air atmosphere.

2.3. Compositions of the CAU-55-Cl samples

The compositions of the CAU-55 sample with different defect concentrations (Table S3) were derived under consideration of the crystal structure of CAU-55, the thermogravimetric measurements and the results of the elemental analyzes. The results of the elemental analysis and weight losses determined by thermogravimetric measurements, as well as a comparison with the theoretical values assuming the proposed chemical compositions, are given in Table 2 of the main article.

Table S3. Proposed chemical compositions and the corresponding molar weight of the CAU-55-Cl samples.

CAU-55-Cl sample	proposed chemical compositions	**M** (g/mol)
**0.5-4-4 (A2)**	$[Al_{24}(OH)_{56}(O_2CCH_3)_{12}]Cl_4 \cdot 29\ H_2O$	2972.8
**1.0-4-4 (C2)**	$[Al_{24}(OH)_{56}(O_2CCH_3)_{12}]Cl_4 \cdot 25\ H_2O$	2900.7
**2.0-4-4 (E2)**	$[Al_{24}(OH)_{56}(O_2CCH_3)_{12}]Cl_4 \cdot 14\ H_2O$	2702.5
**1.0-5-4 (C3)**	$[Al_{24}(OH)_{56}(O_2CCH_3)_{12}]Cl_4 \cdot 17\ H_2O$	2746.6
**1.0-5-6 (D1)**	$[Al_{24}(OH)_{56}(O_2CCH_3)_{12}]Cl_4 \cdot 25\ H_2O$	2900.7

2.4. Scanning electron microscopy (SEM)

Scanning electron microscopy measurements were carried out using a JEOL JSM-7000F microscope operated at 2 kV for two representative samples of CAU-55-Cl (Figure S9).

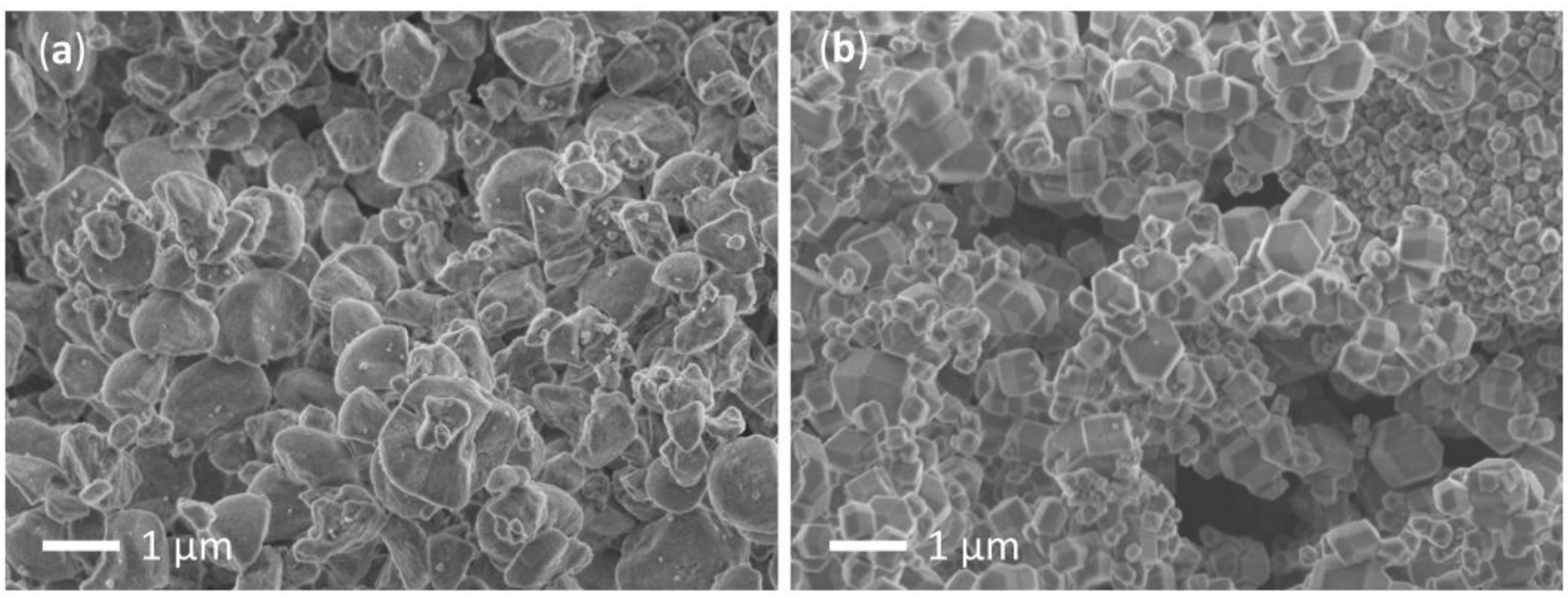

Figure S9. Scanning electron micrographs of two representative samples of CAU-55-Cl with (sample 1-5-5 (C5), (**a**)) and without cluster defects (sample 2-4-4 (E2), (**b**)).

3. Sorption properties

Additional N_2, CO_2 and Ar gas and water vapor sorption measurements (Figures S10 – S15, Figures S17 – S18, Figure S24) were carried out using a BEL Japan Inc. BELSORP-max with nitrogen gas at 77 K and water vapor at 298 K. The CO_2 gas sorption measurements were carried out at 273 and 298 K. Prior to the measurements, the samples were activated for 3 h at elevated temperatures (120 °C) under reduced pressure ($p < 10^{-2}$ kPa). Subsequently, the integrity and the long-range order of the samples after thermal activation and the sorption measurements was confirmed by PXRD measurements after the sorption experiments (Figures S19 – S23).

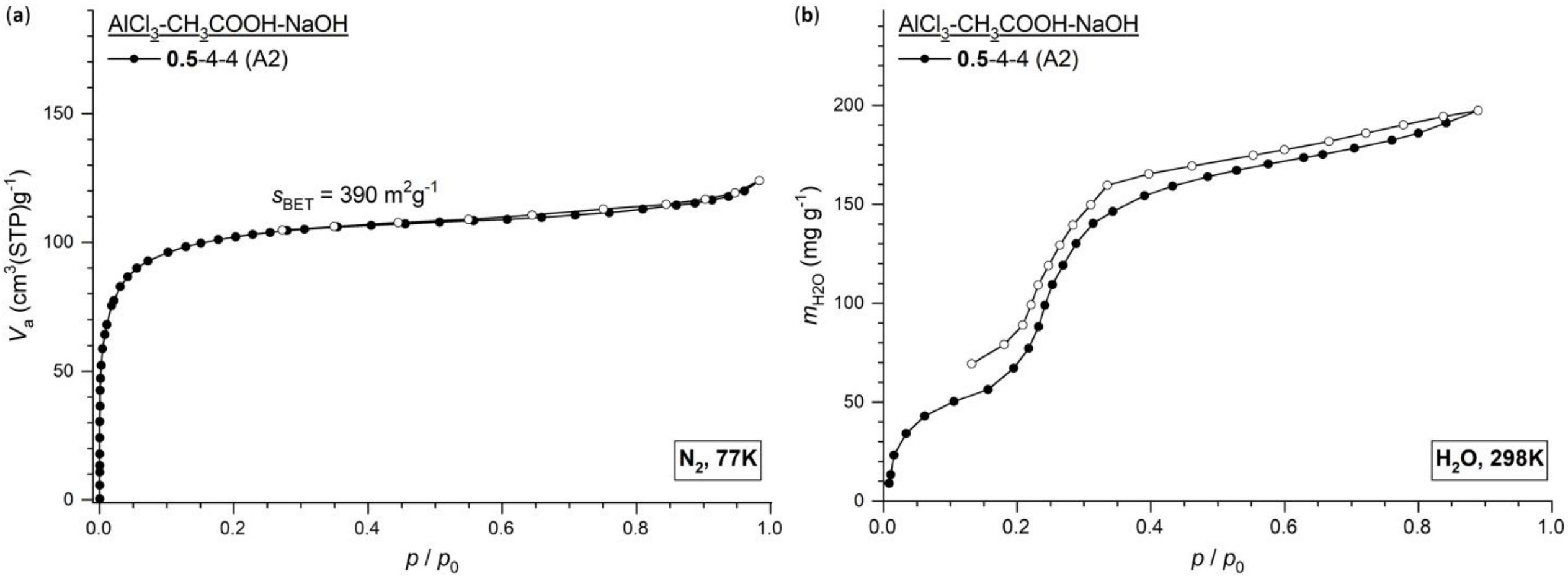

Figure S10. N_2 sorption (**a**) and H_2O sorption (**b**) isotherm of CAU-55-Cl (0.5-4-4 (A2)) collected at 77 K and 298 K, respectively. Prior to the measurements the samples were activated for 3 h at 120 °C under reduced pressure ($p < 10^{-2}$ kPa). The apparent specific surface area was determined from the N_2 sorption isotherm employing the methodology by Rouquerol.[4]

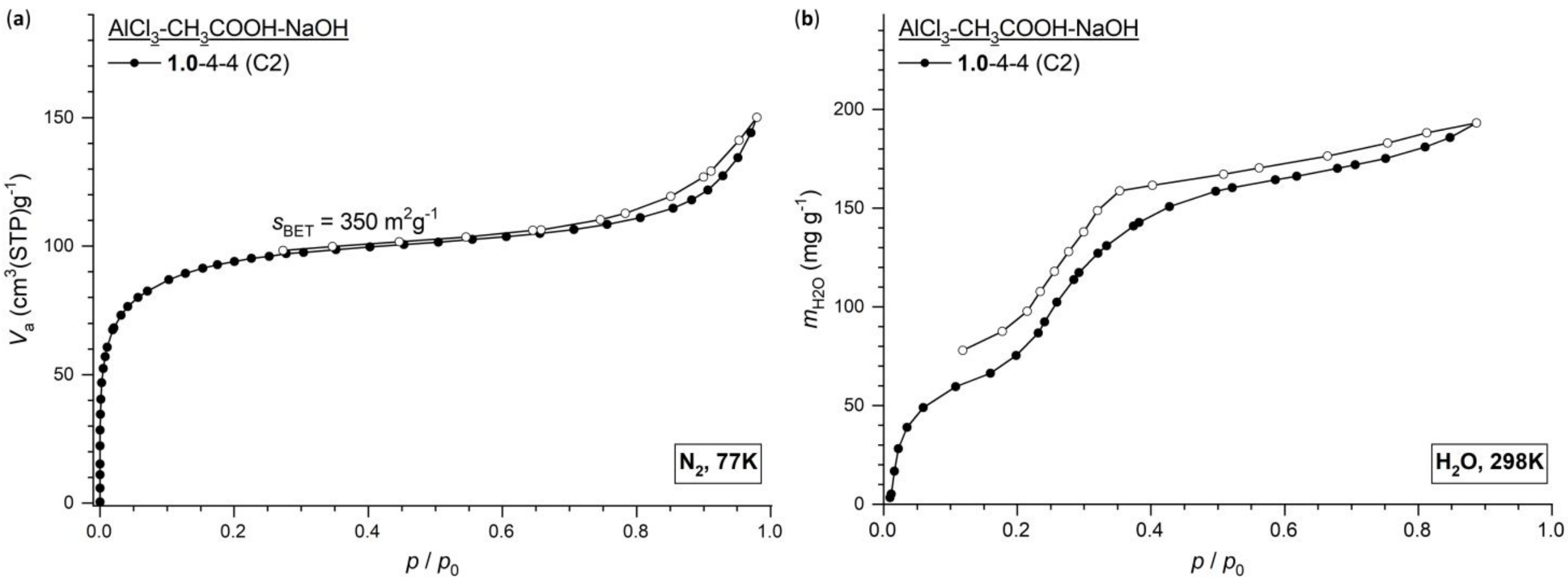

Figure S11. N_2 sorption (**a**) and H_2O sorption (**b**) isotherm of CAU-55-Cl (1.0-4-4 (C2)) collected at 77 K and 298 K, respectively. Prior to the measurements the samples were activated for 3 h at 120 °C under reduced pressure ($p < 10^{-2}$ kPa). The apparent specific surface area was determined from the N_2 sorption isotherm employing the methodology by Rouquerol.[4]

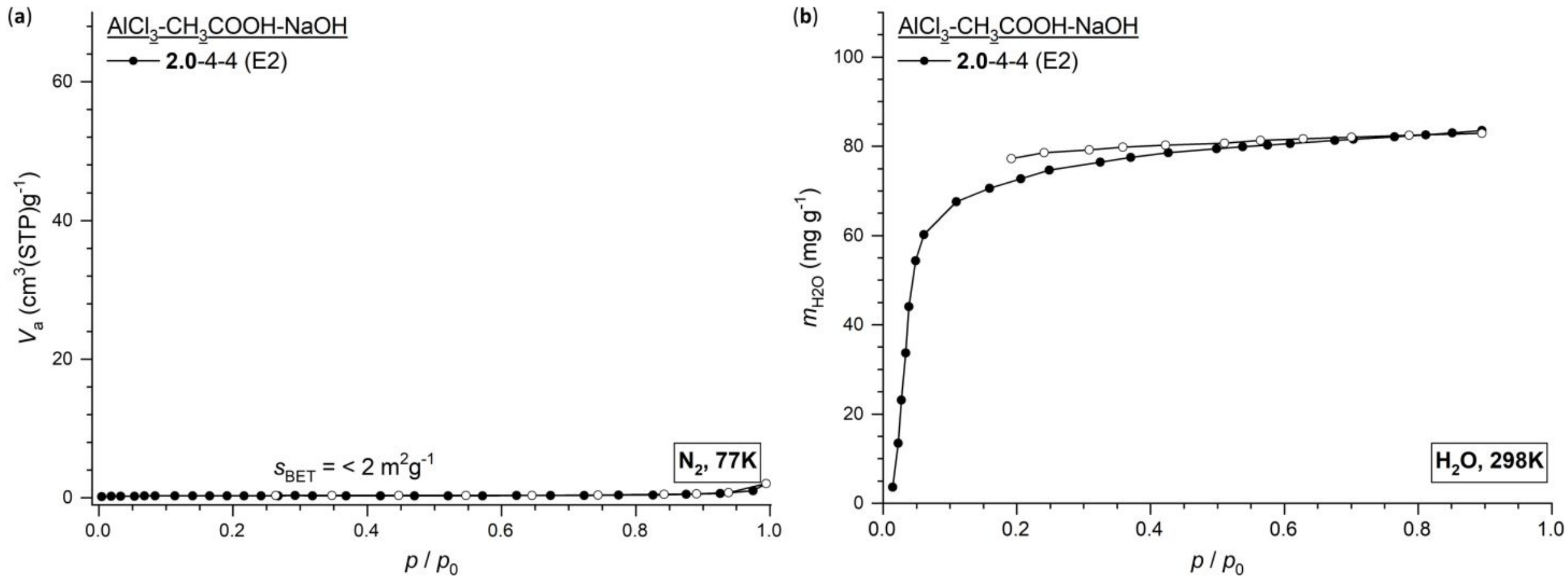

Figure S12. N_2 sorption (**a**) and H_2O sorption (**b**) isotherm of CAU-55-Cl (2.0-4-4 (E2)) collected at 77 K and 298 K, respectively. Prior to the measurements the samples were activated for 3 h at 120 °C under reduced pressure ($p < 10^{-2}$ kPa). The apparent specific surface area was determined from the N_2 sorption isotherm employing the methodology by Rouquerol.[4]

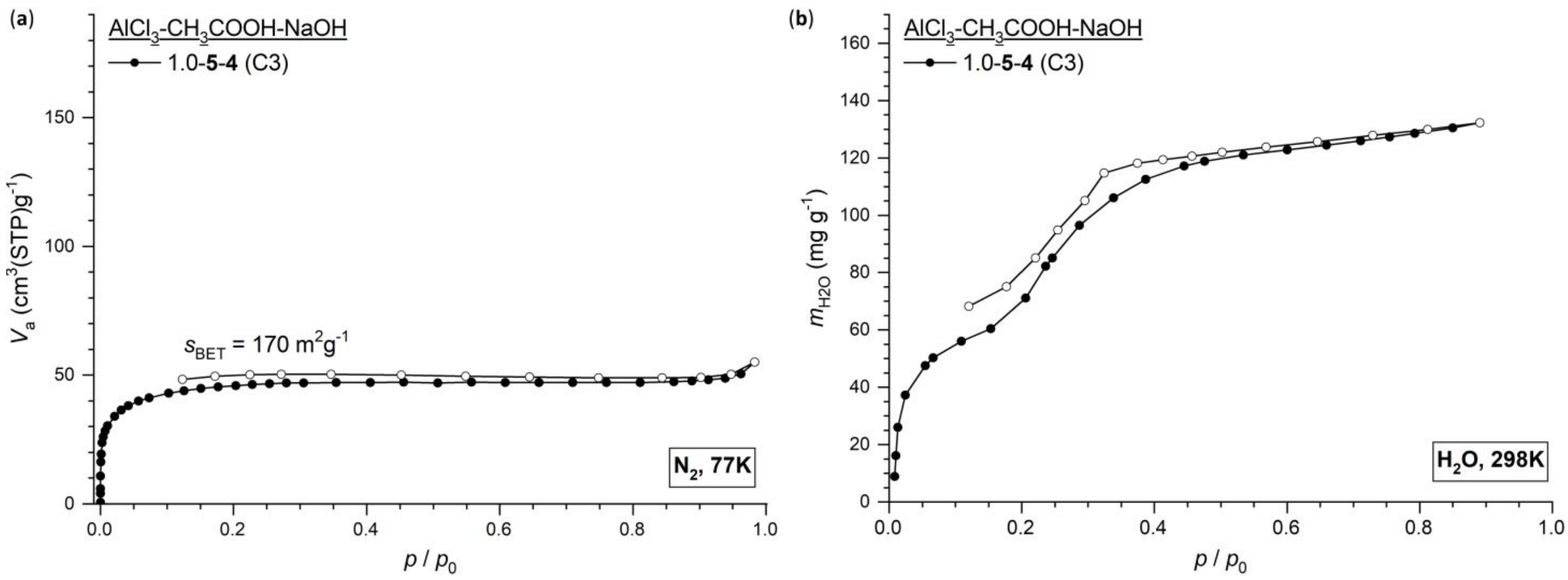

Figure S13. N_2 sorption (**a**) and H_2O sorption (**b**) isotherm of CAU-55-Cl (1.0-5-4 (C3)) collected at 77 K and 298 K, respectively. Prior to the measurements the samples were activated for 3 h at 120 °C under reduced pressure ($p < 10^{-2}$ kPa). The apparent specific surface area was determined from the N_2 sorption isotherm employing the methodology by Rouquerol.[4]

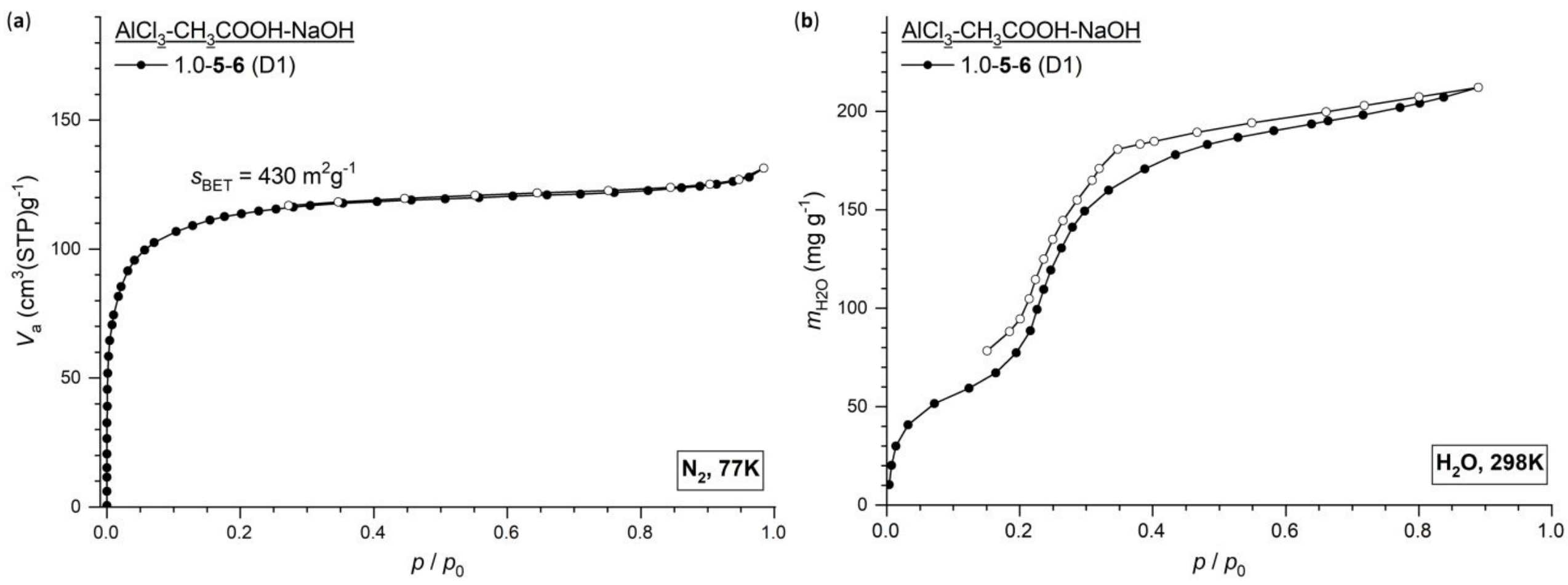

Figure S14. N_2 sorption (**a**) and H_2O sorption (**b**) isotherm of CAU-55-Cl (0.5-5-6 (D1)) collected at 77 K and 298 K, respectively. Prior to the measurements the samples were activated for 3 h at 120 °C under reduced pressure ($p < 10^{-2}$ kPa). The apparent specific surface area was determined from the N_2 sorption isotherm employing the methodology by Rouquerol.[4]

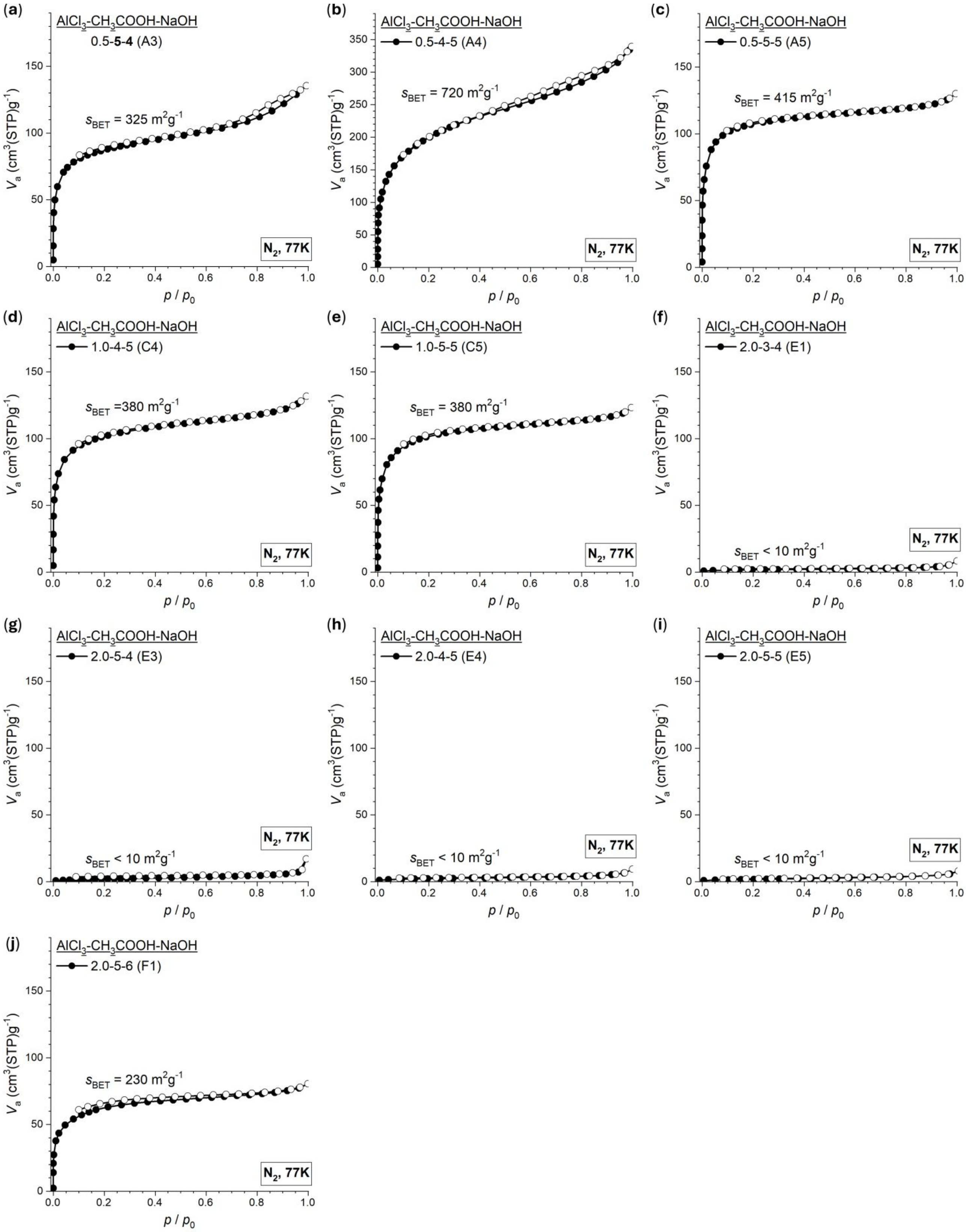

Figure S15. Additional N_2 sorption isotherms of CAU-55-Cl collected at 77 K. Prior to the measurements the samples were activated for 3 h at 120 °C under reduced pressure ($p < 10^{-2}$ kPa). The apparent specific surface areas were determined from the N_2 sorption isotherm employing the methodology by Rouquerol.[4]

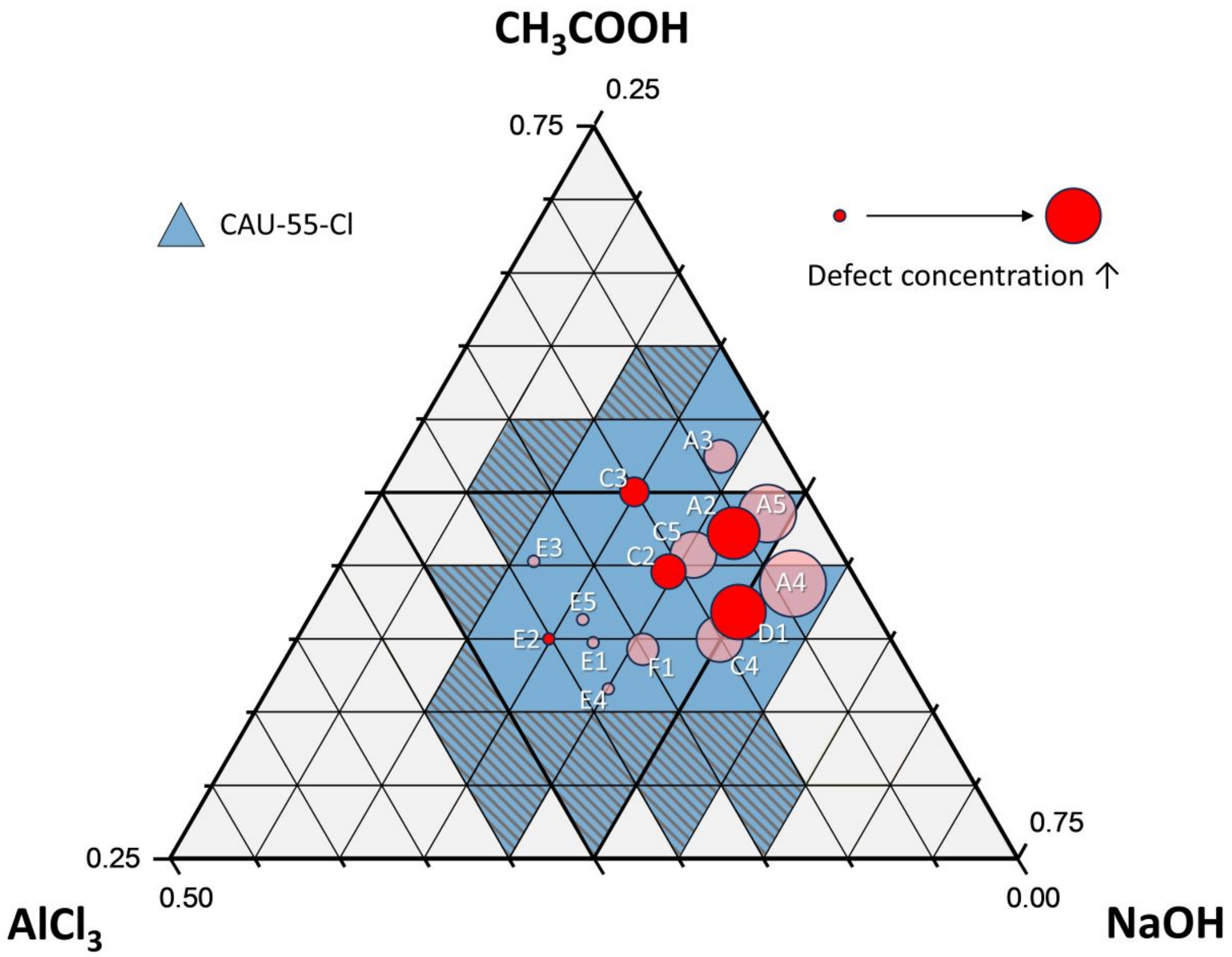

Figure S16. Ternary crystallization diagram of CAU-55-Cl observed in the chemical system $AlCl_3$/CH_3COOH/NaOH for reactions carried out at 100 °C. The blue triangles indicate the parameter spaces in which CAU-55-Cl was obtained and grey-hatched triangles represent parameter spaces where CAU-55 was obtained together with other crystalline phases as a phase mixture (see Table S1.1). The samples are labelled according to the molar ratios used in the synthesis: A2 (0.5-4-4), A3 (0.5-5-4), A4 (0.5-4-5), A5 (0.5-5-5), C2 (1-4-4), C3 (1-5-4), C4 (1-4-5), C5 (1-5-5), D1 (1-5-6), E1 (2-3-4), E2 (2-4-4), E3 (2-5-4), E4 (2-4-5), E5 (2-5-5), F1 (2-5-6). The corresponding defect concentrations are illustrated by red dots of different sizes (indicated by the apparent specific surface areas of the samples obtained from N_2 sorption measurements).

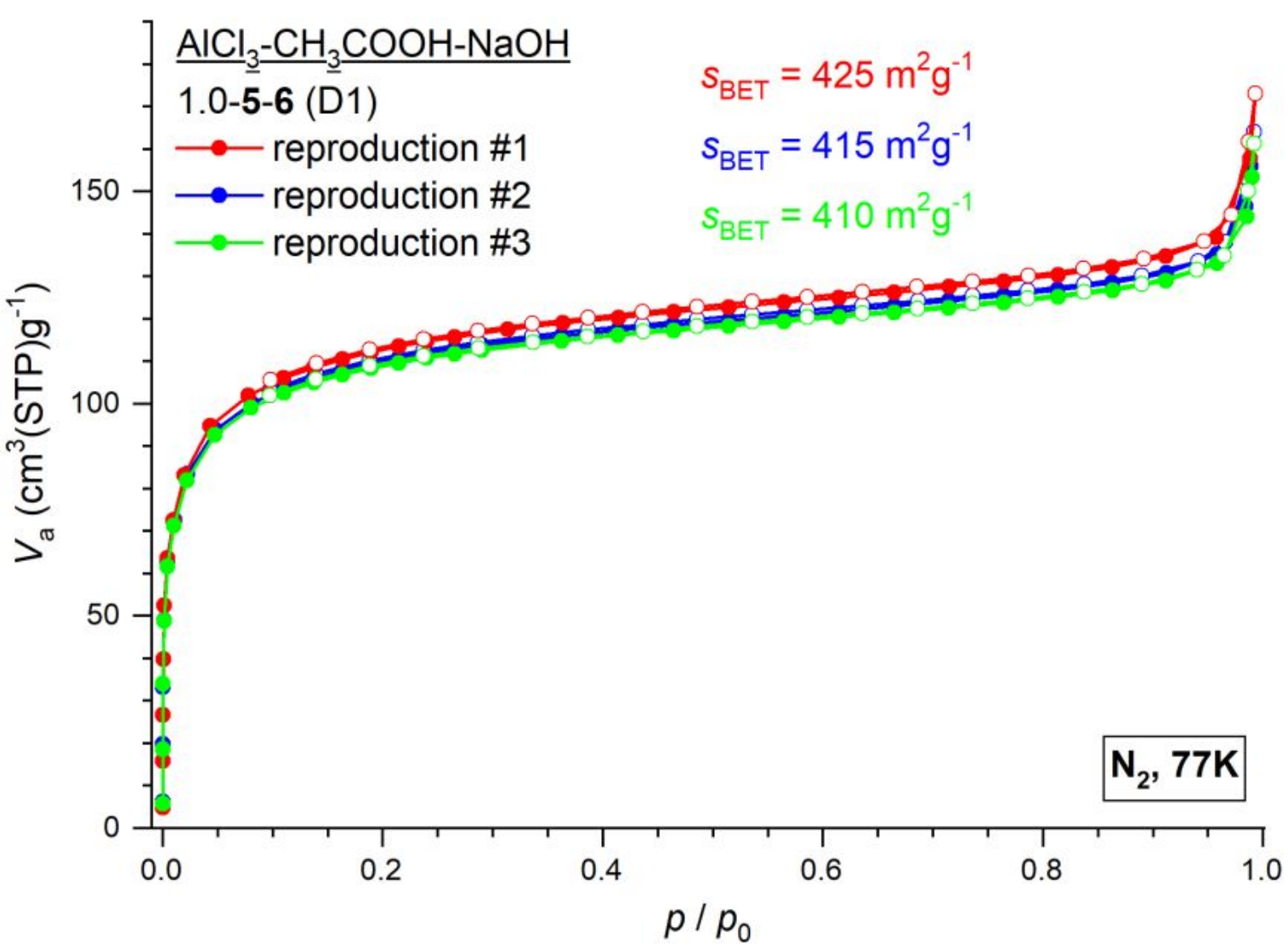

Figure S17. Comparison of the N_2 sorption isotherms collected at 77 K of three independently synthesized samples of CAU-55-Cl (0.5-5-6 (D1)) demonstrating the reproducibility of CAU-55-Cl with specific defect concentrations depending on the molar ratios of the starting materials used for the synthesis. Prior to the measurements the samples were activated for 3 h at 120 °C under reduced pressure ($p < 10^{-2}$ kPa). The apparent specific surface areas were determined from the N_2 sorption isotherm employing the methodology by Rouquerol.[4]

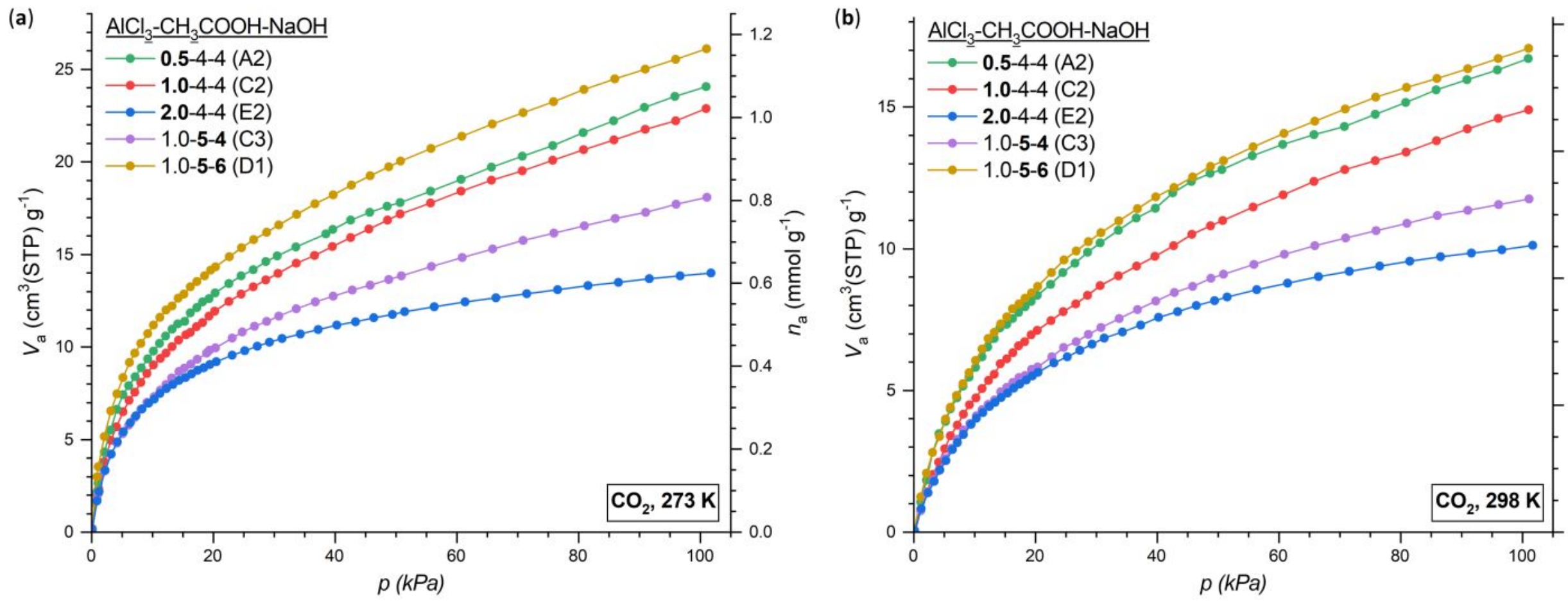

Figure S18. CO_2 sorption isotherms of the different samples of CAU-55-Cl with different defect concentrations. The sorption measurements were carried out at 273 K (**a**) and 298 K (**b**), respectively. Prior to the measurements the samples were activated for 3 h at 120 °C under reduced pressure ($p < 10^{-2}$ kPa).

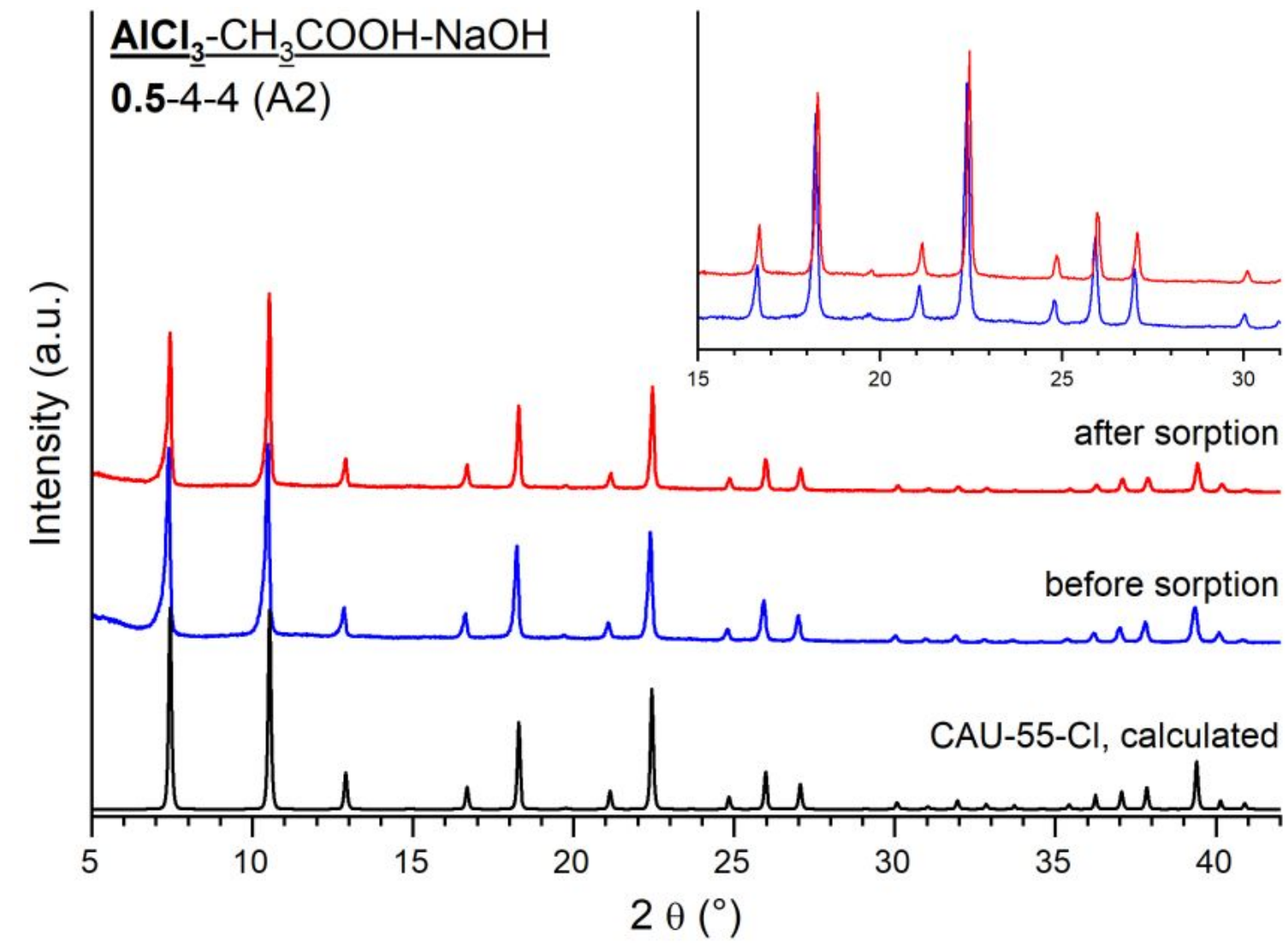

Figure S19. PXRD patterns of CAU-55-Cl (0.5-4-4(A2)) before and after N_2, CO_2 and H_2O sorption experiments.

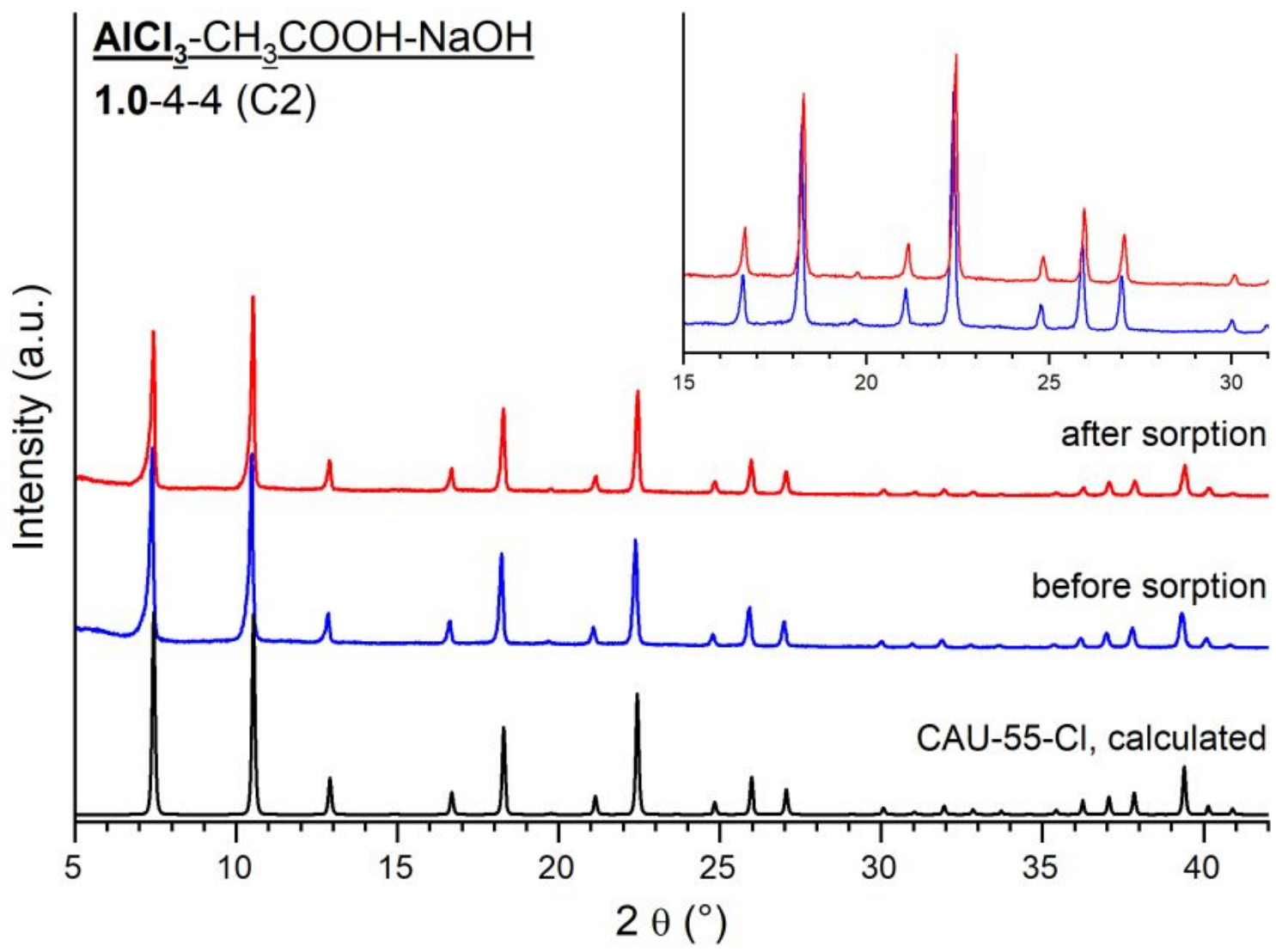

Figure S20. PXRD patterns of CAU-55-Cl (1-4-4(C2)) before and after N_2, CO_2 and H_2O sorption experiments.

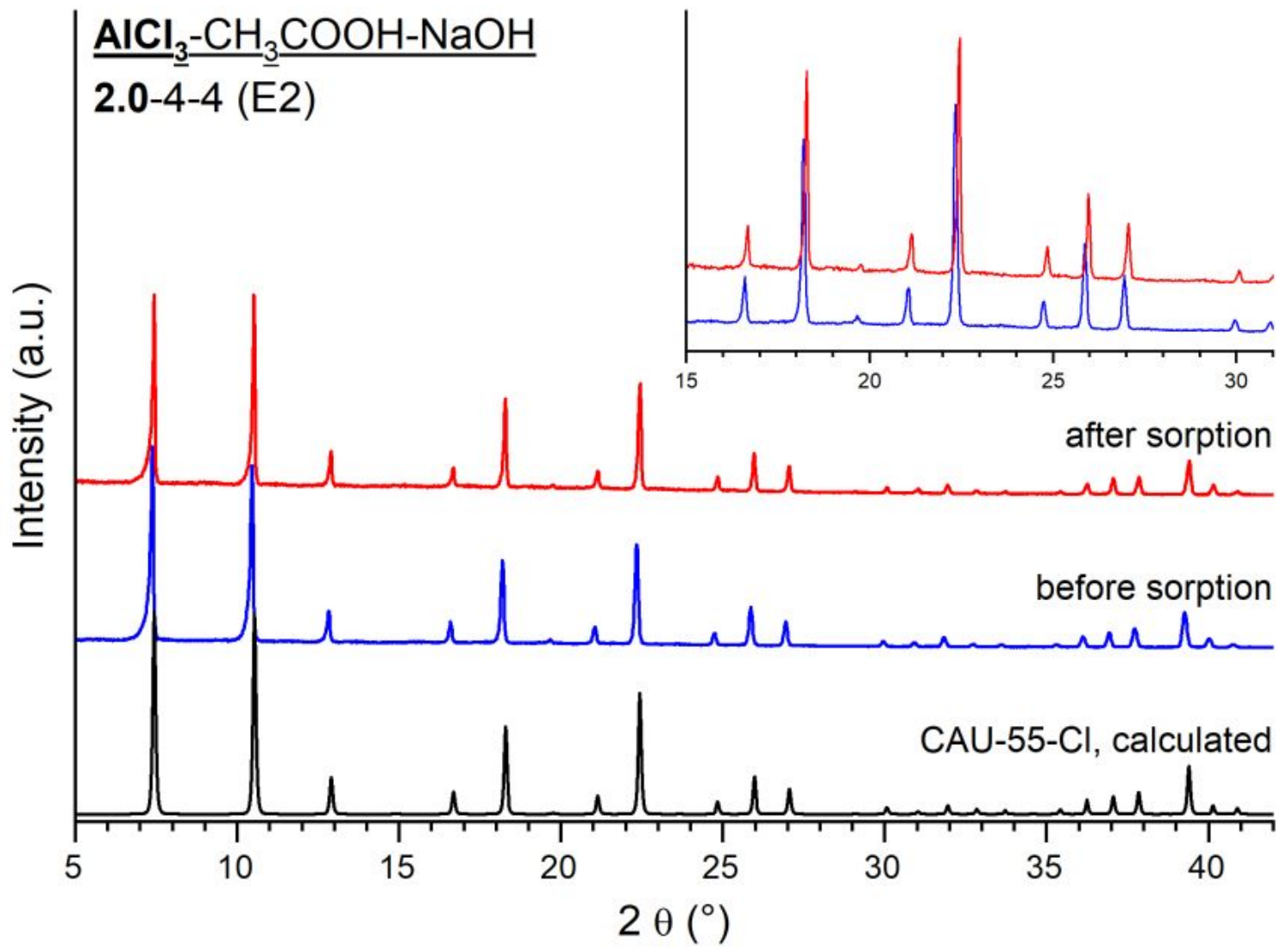

Figure S21. PXRD patterns of CAU-55-Cl (2-4-4(E2)) before and after N_2, CO_2 and H_2O sorption experiments.

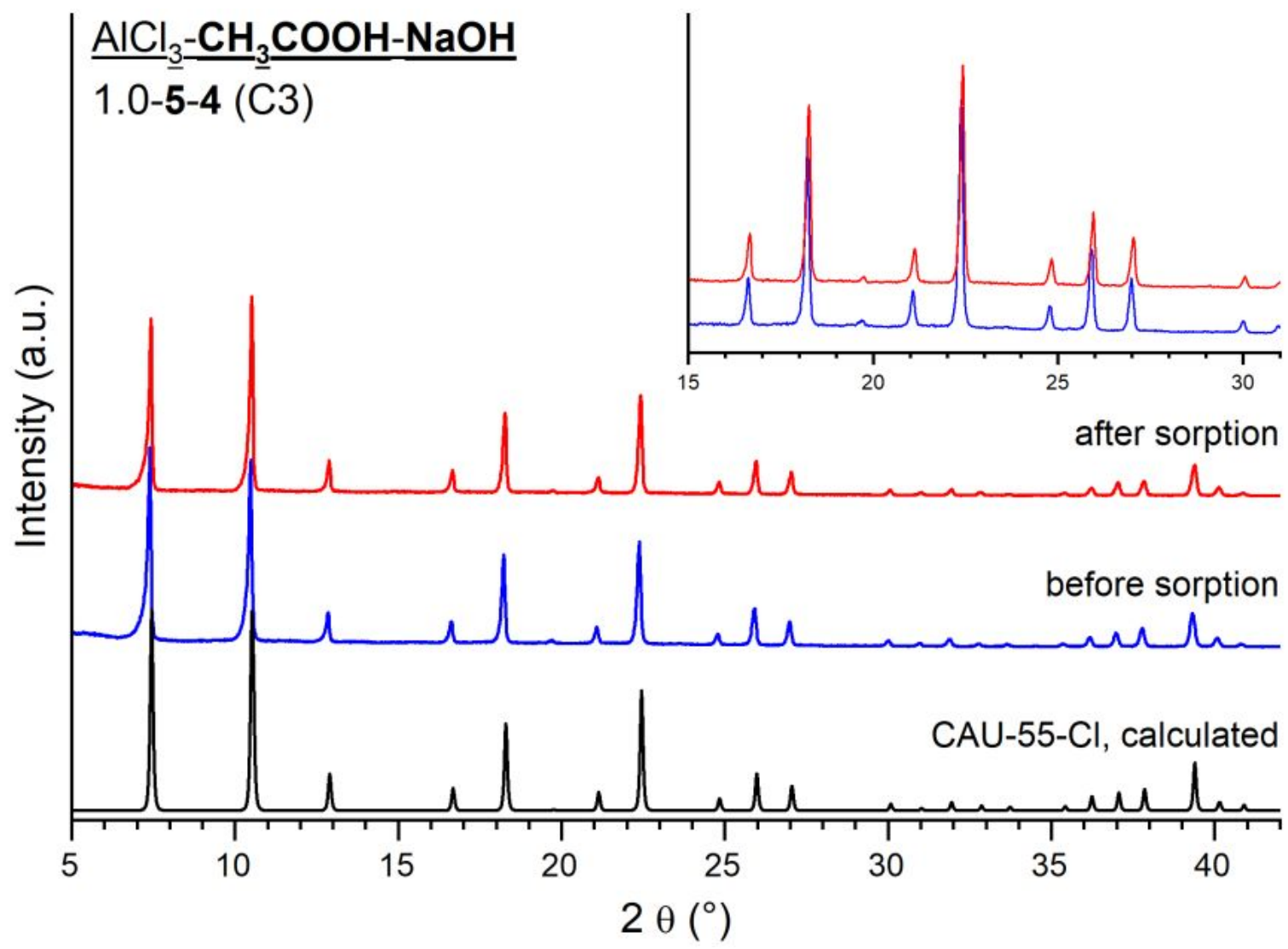

Figure S22. PXRD patterns of CAU-55-Cl (1-5-4(C3)) before and after N_2, CO_2 and H_2O sorption experiments.

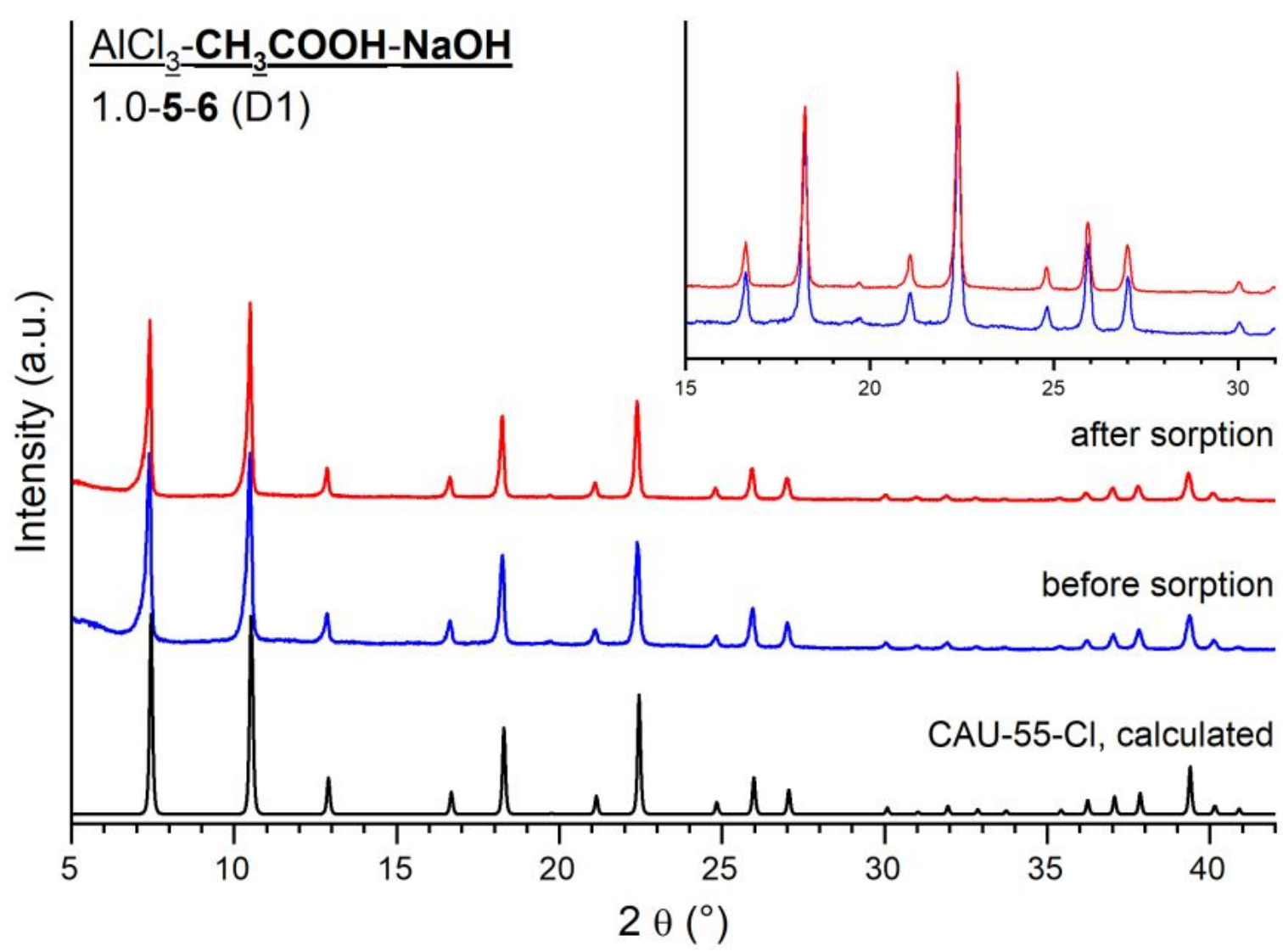

Figure S23. PXRD patterns of CAU-55-Cl (1-5-6(D1)) before and after N_2, CO_2 and H_2O sorption experiments.

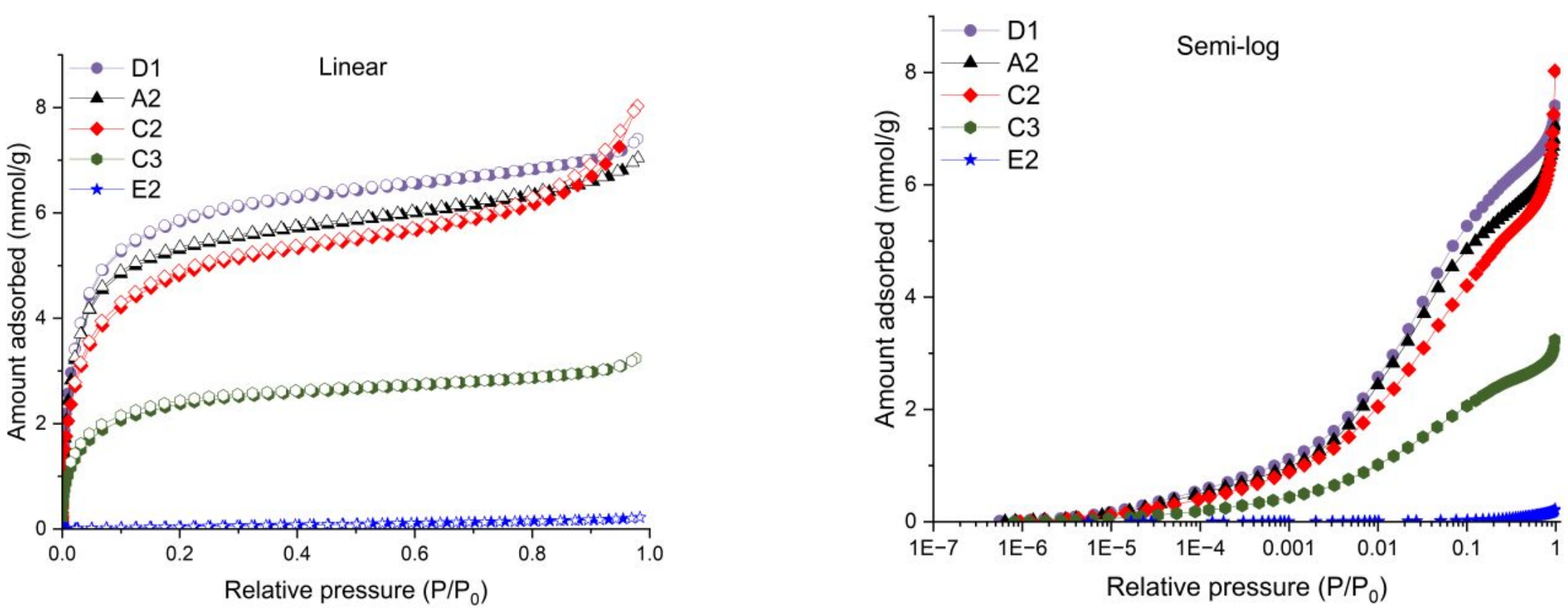

Figure S24. Linear (left) and semi-log (right) Ar sorption isotherms of the different samples of CAU-55-Cl with different defect concentrations, i.e. D1: 1-5-6; A2: 0.5-4-4; C2: 1-4-4; C3: 1-5-4; E2: 2-4-4. The sorption measurements were carried out at 87 K over a wide range of relative pressure from $p/p_0 = 10^{-6}$ to 1. Prior to the measurements the samples were activated for 3 h at 120 °C under reduced pressure ($p < 10^{-2}$ kPa).

4. Computational Details

4.1. Force field definitions

Table S4. Force field parameters for the porous salt CAU-55 and Ar atoms.

Atom	σ (Å)	ε (K)	Force Field
Al	4.0082	0.0	UFF[5]
C	3.4730	47.857	Dreiding[6]
O	3.1180	30.193	Dreiding[6]
H	2.8644	0.0	Dreiding[6]
Cl	3.516	114.232	UFF[5]
Ar	3.405	119.76	Ref.[7,8]

4.2. Graph network porosity analysis

Graph network analysis of defective models. The dots represent network nodes within the accessible porosities, highlighting intra-cluster voids and missing-cluster defects. Three-dimensional models were generated by spatially combining the nodes into a 3D network. Additionally, .gif files showing all frames of the pore-node distribution, as well as .obj files of the corresponding 3D models, are provided S.I. at https://github.com/sabrinagri/supporting-info.

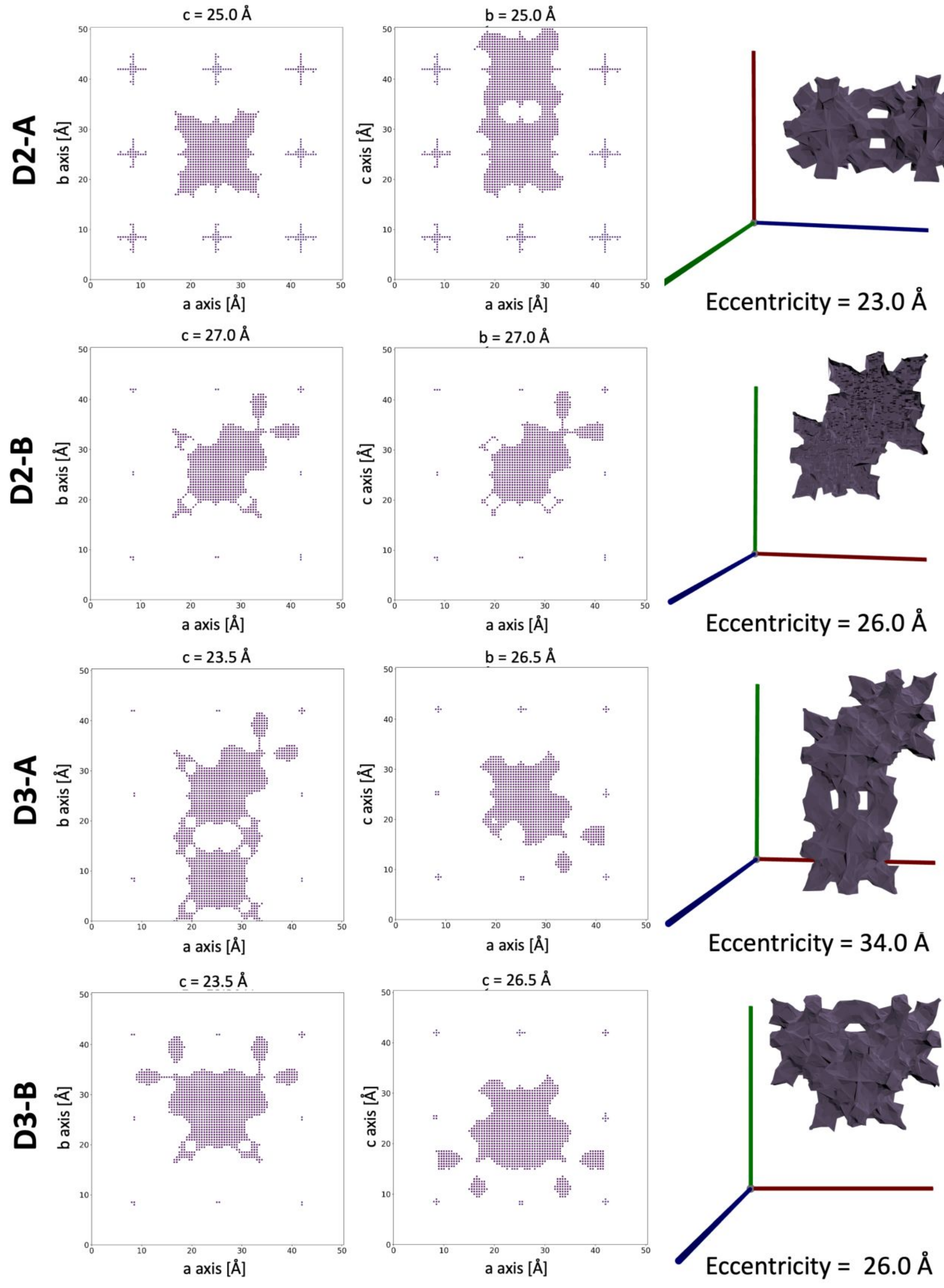

Figure S25. Graph network analysis of isolated defective models D2 and D3. Slab plots along reference axes illustrate the distribution of network nodes within the porosities, while the three-dimensional models provide a visual representation of the defects

4.3. GCMC simulated adsorption isotherms and adsorption mechanism

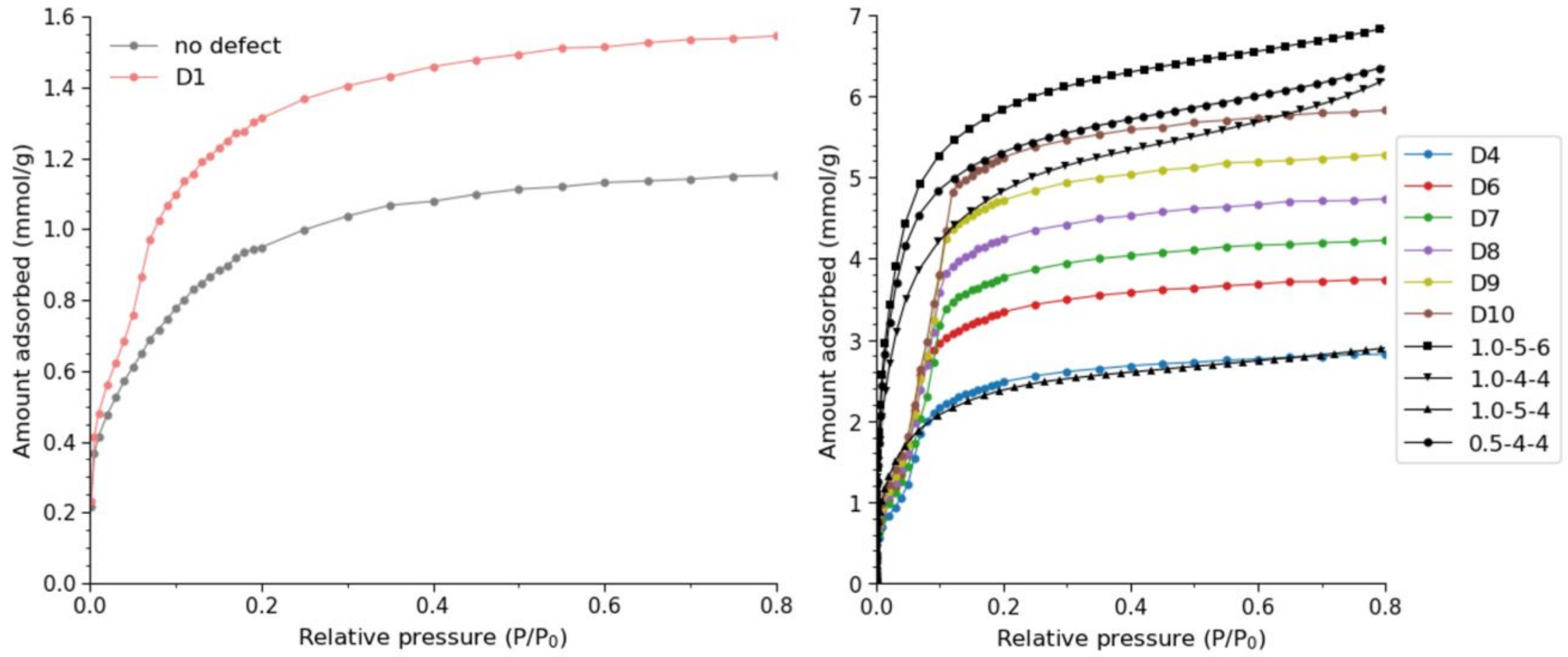

Figure S26. (a) GCMC adsorption isotherms for the non-defective CAU-55-Cl model and the D1 model (single missing-cluster defect). **(b)** Comparison between simulated (colored curves, models D4–D10) and experimental (black symbols) argon adsorption isotherms for selected samples.

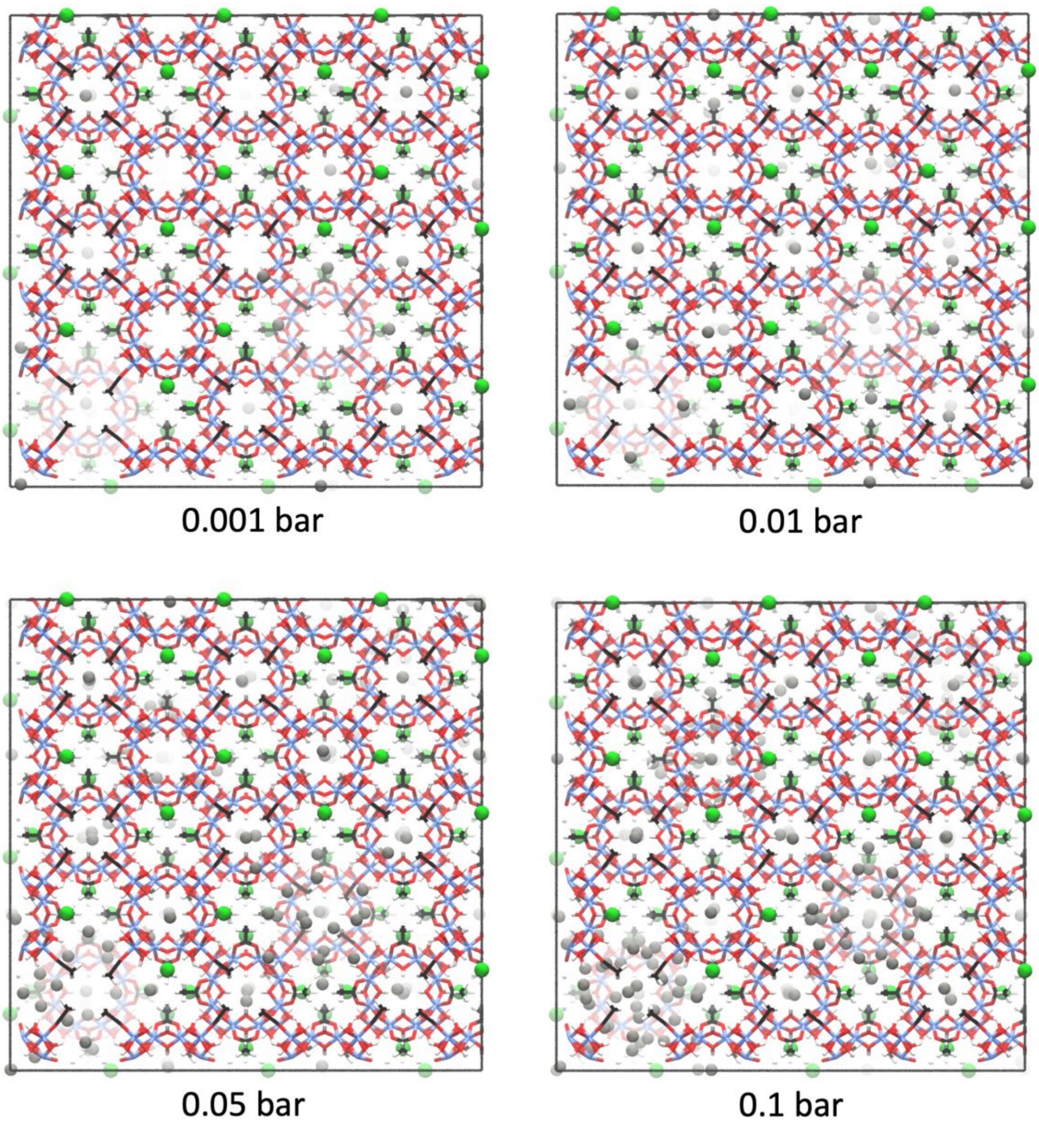

Figure S27. Adsorption mechanism of argon in defective CAU-55-Cl. GCMC snapshots illustrating the adsorption of Ar in the D4 model (4 out of 54 clusters missing) of CAU-55-Cl at various pressures. Atom color code: Al (purple), O (red), C (black), H (white), Cl (green) and Ar (grey).

5. References

1 G. Socrates, *Infrared and Raman Characteristic Group Frequencies: Tables and Charts*, J. Wiley and Sons, Chichester, 3rd edn., 2010.

2 J.-J. Max and C. Chapados, *J. Phys. Chem. A*, 2004, **108**, 3324–3337.

3 M. Ibrahim and E. Koglin, *Acta Chim. Slov.*, 2004, **51**, 453–460.

4 J. Rouquerol, P. Llewellyn and F. Rouquerol, 2007, pp. 49–56.

5 A. K. Rappé, C. J. Casewit, K. S. Colwell, W. A. Goddard and W. M. Skiff, *J. Am. Chem. Soc.*, 1992, **114**, 10024–10035.

6 S. L. Mayo, B. D. Olafson and W. A. Goddard, *J. Phys. Chem.*, 1990, **94**, 8897–8909.

7 K. Potier, K. Ariskina, A. Obliger and J.-M. Leyssale, *Langmuir*, 2023, **39**, 9384–9395.

8 B. Coasne and R. J.-M. Pellenq, *J. Chem. Phys.*, 2004, **120**, 2913–2922.
